# Dual Roles for Membrane Association of Drosophila Axin in Wnt Signaling

**DOI:** 10.1371/journal.pgen.1006494

**Published:** 2016-12-13

**Authors:** Zhenghan Wang, Ofelia Tacchelly-Benites, Eungi Yang, Yashi Ahmed

**Affiliations:** Department of Molecular and Systems Biology and the Norris Cotton Cancer Center, Geisel School of Medicine at Dartmouth College, Hanover, NH, United States of America; University of Michigan, UNITED STATES

## Abstract

Deregulation of the Wnt signal transduction pathway underlies numerous congenital disorders and cancers. Axin, a concentration-limiting scaffold protein, facilitates assembly of a “destruction complex” that prevents signaling in the unstimulated state and a plasma membrane-associated “signalosome” that activates signaling following Wnt stimulation. In the classical model, Axin is cytoplasmic under basal conditions, but relocates to the cell membrane after Wnt exposure; however, due to the very low levels of endogenous Axin, this model is based largely on examination of Axin at supraphysiological levels. Here, we analyze the subcellular distribution of endogenous Drosophila Axin *in vivo* and find that a pool of Axin localizes to cell membrane proximal puncta even in the absence of Wnt stimulation. Axin localization in these puncta is dependent on the destruction complex component Adenomatous polyposis coli (Apc). In the unstimulated state, the membrane association of Axin increases its Tankyrase-dependent ADP-ribosylation and consequent proteasomal degradation to control its basal levels. Furthermore, Wnt stimulation does not result in a bulk redistribution of Axin from cytoplasmic to membrane pools, but causes an initial increase of Axin in both of these pools, with concomitant changes in two post-translational modifications, followed by Axin proteolysis hours later. Finally, the ADP-ribosylated Axin that increases rapidly following Wnt stimulation is membrane associated. We conclude that even in the unstimulated state, a pool of Axin forms membrane-proximal puncta that are dependent on Apc, and that membrane association regulates both Axin levels and Axin’s role in the rapid activation of signaling that follows Wnt exposure.

## Author Summary

Axin is a scaffold protein with essential roles in Wnt signal transduction. In the classical model, the transition from the unstimulated to stimulated state is thought to be achieved by recruitment of Axin from cytosol to plasma membrane. We find that a pool of endogenous Drosophila Axin is localized in puncta juxtaposed with the cell membrane even under basal conditions and is targeted for degradation by the ADP-ribose polymerase Tankyrase. Wnt stimulation initially results in increased Axin levels in both the cytosolic and membrane pools, which may enhance the robust activation of signaling.

## Introduction

The Wnt/Wingless signal transduction pathway directs fundamental cellular processes during animal development and tissue homeostasis, whereas Wnt pathway deregulation results in numerous cancers and congenital disorders [1, 2]. In the unstimulated state, the concentration-limiting scaffold protein Axin facilitates assembly of a cytoplasmic “destruction complex” that includes the tumor suppressor Adenomatous polyposis coli (Apc) as well as glycogen synthase kinase 3 (GSK3) and targets the transcriptional activator β-catenin for proteasomal degradation. Binding of Wnt ligands to their transmembrane co-receptors LRP6/Arrow and Frizzled induces rapid phosphorylation of the intracellular tail of LRP6, creating binding sites for Axin [3–6]. The consequent recruitment of Axin, GSK3, and the cytoplasmic component Dishevelled to LRP6 and Frizzled promotes assembly of an activation complex termed the “signalosome” [7]. Axin is thought to facilitate signaling by acting as a scaffold for the signalosome [3, 5] and by promoting LRP6 phosphorylation following Wnt stimulation [4], although the initial phosphorylation of LRP6 may occur independently of Axin [8]. Signalosome assembly results in β-catenin stabilization and the transcriptional regulation of Wnt pathway target genes [3, 4, 9].

The levels of Axin under basal conditions are very low [10, 11], and regulated by the ADP-ribose polymerase Tankyrase (Tnks). Tnks-mediated ADP-ribosylation targets Axin for ubiquitin-dependent proteasomal degradation [12–14]. The role of Tnks in controlling Axin levels is conserved in Drosophila [15–18]. Due to functional redundancy in vertebrate Tnks homologs [19], the *in vivo* settings in which mammalian Tnks promotes Wnt signaling remain uncertain, but it is known that in Drosophila, the requirement for Tnks is context-specific, as Tnks is dispensable for many Wingless-dependent developmental processes [15, 17]. However, in the adult Drosophila intestine, Tnks is essential for target gene activation within regions where Wingless is present at relatively low concentration and promotes the Wingless-dependent regulation of midgut stem cell proliferation [17, 20]. Furthermore, when endogenous Axin levels are increased by only two-fold, Tnks is required for Wingless-dependent cell fate specification in the embryonic epidermis [18]. In addition, Tnks-mediated ADP-ribosylation of Axin promotes not only Axin proteolysis in the unstimulated state, but also the rapid transition in Axin activity that follows Wnt stimulation and the interaction of Axin with phospho-LRP6, which is a key step in the activation of signaling [18].

In the classical model, Axin localizes in the cytoplasm under basal conditions, but relocates to the plasma membrane following Wnt stimulation [7, 21]. However analysis of Axin regulation under physiological conditions has been impeded by the very low levels of endogenous Axin. Therefore, previous *in vivo* work regarding the relocation of Axin that follows Wnt stimulation was based largely on overexpression of Axin to levels that completely inhibited Wnt signaling in Drosophila embryos, thus disrupting its physiological regulation [21, 22]. In these studies, overexpressed Axin was described as “dots” that localized either throughout the cytoplasm in the unstimulated state or primarily at the plasma membrane after Wingless stimulation. Based on these findings, the authors concluded that Wingless exposure induces the bulk relocation of Axin from cytoplasm to plasma membrane. However, the Axin-GFP fusion protein used in these studies was not only highly overexpressed but also aberrantly stabilized; this Axin-GFP abrogated Wnt signaling and was refractory to the degradation of Axin that occurs several hours after Wnt stimulation in Drosophila [18, 23] and vertebrate cells [8, 24–26].

Here, we investigate the regulation of endogenous Drosophila Axin *in vivo*. We find that even in the unstimulated state, a pool of Axin is localized in puncta at the cell periphery through association with the plasma membrane and/or vesicles just proximal to the membrane. Axin’s localization at these peripheral puncta is independent of Wingless stimulation, but dependent on Apc. Furthermore, membrane association increases the ADP-ribosylation of Axin and thereby promotes Tnks-mediated proteasomal degradation under basal conditions. Moreover, we find no evidence for the bulk redistribution of Axin from cytosolic to membrane-associated pools following Wingless stimulation *in vivo* or in cultured embryonic cells. Instead, we find that Wingless exposure initially results in increased levels of both cytoplasmic and membrane-associated Axin accompanied by changes in two post-translational modifications, followed by Axin proteolysis hours later. Importantly, Wingless stimulation induces a preferential increase in the pool of ADP-ribosylated Axin associated with the membrane. As ADP-ribosylation promotes the interaction of Axin with phospho-LRP6 [18], the increased levels of membrane-associated ADP-ribosylated Axin may facilitate rapid and robust Wnt pathway activation through both local enrichment at the membrane and increased interaction with LRP6. We conclude that the formation of membrane-proximal Axin puncta, some of which may represent the sites of the destruction complex, depends on Apc, and that membrane association both regulates Axin levels and leaves Axin poised for rapid activation of the Wnt pathway.

## Results

### Axin is localized in cell membrane-proximal puncta in the unstimulated state

**Fig 1:**
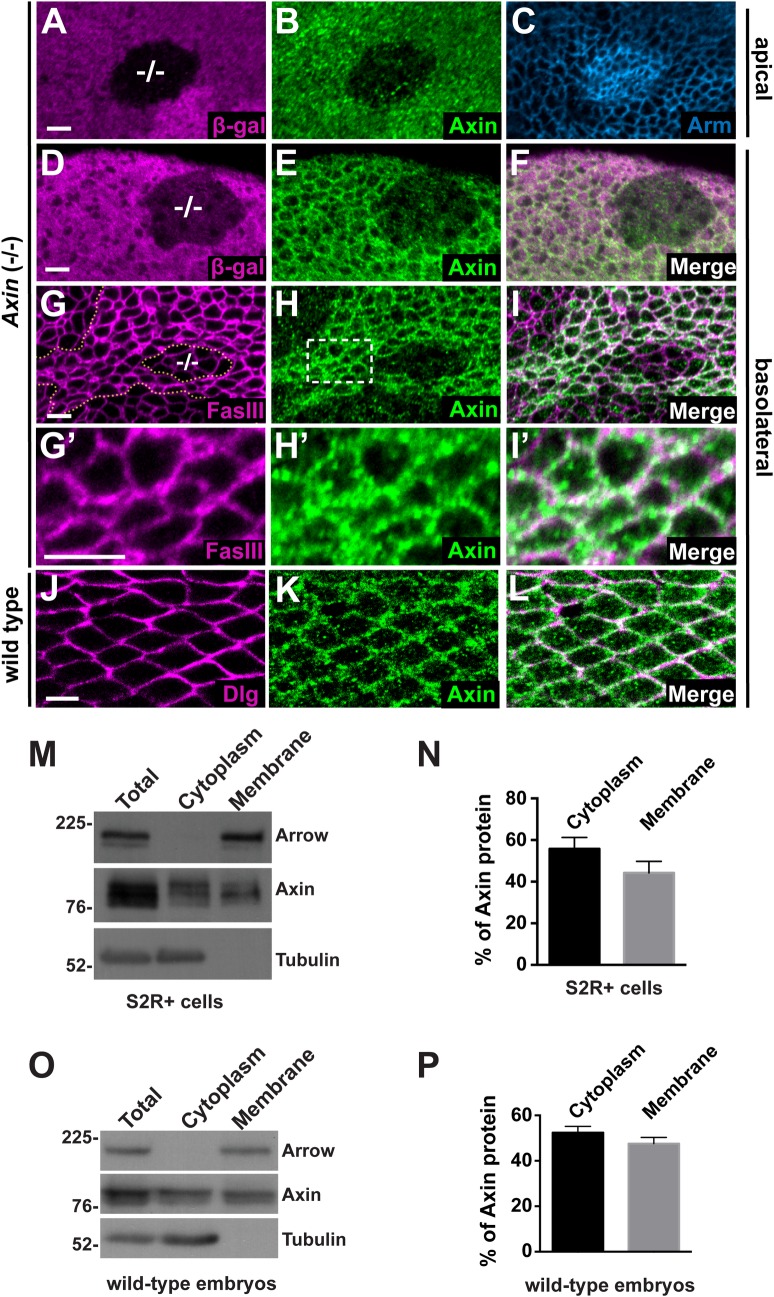
Axin is localized at cell membrane-proximal puncta independently of Wingless pathway activation (A-I) Confocal images of third instar larval wing imaginal discs stained with antibodies indicated at bottom right; genotypes at left margin. (A-C) Wing disc stained with β-gal (A, magenta), Axin (B, green), and Arm (C, blue) antibodies. *Axin*^*18*^ null mutant clones (marked by the absence of β-gal, -/- in A) demonstrate the specificity of the Axin antibody. Armadillo marks the adherens junctions, which are present at the boundary between the apical and basolateral membrane, and also accumulates in the cytoplasm in *Axin* mutant clones. At this apical level, Axin staining is diffuse in the cytoplasm of all cells. (D-I) Axin staining at basolateral levels in the wing disc. *Axin*^*18*^ null mutant clones are marked by the absence of β-gal (D) or by dashed line (G). At this level, Axin antibody reveals specific staining that partially overlaps the basolateral membrane marker Fas III (I). Higher magnification views of the boxed area in (H) reveals endogenous Axin is present in puncta at or near the plasma membrane (G’-I’). Images were taken at the periphery of the wing discs. (J-L) Wild-type pupal wing (~28 hrs after pupa formation) double labeled with α-Dlg (J) and α-Axin (K). Endogenous Axin is also present in puncta proximal to the cell membrane in pupal wing (L). (M) Subcellular fractionation of lysates from S2R+ cells. The total lysates, cytoplasmic, and membrane fractions were analyzed by SDS-PAGE. Immunoblotting with Axin antibody revealed that Axin is present in both the cytoplasmic and membrane fractions. The efficiency of the fractionation was assayed by the presence of Arrow and Tubulin, membrane and cytoplasmic markers, respectively. (N) Quantification of the distribution of endogenous Axin in S2R+ cells. Results were obtained from four independent experiments, with a representative blot shown in (M). Values indicate mean ± SD. (O) Subcellular fractionation of lysates from 0–2.5 hour wild-type embryos. (P) Quantification of the distribution of endogenous Axin in 0–2.5 hour wild-type embryos. Results were obtained from four independent experiments, with a representative blot shown in (O). Values indicate mean ± SD. Scale bar: 5μm.

To characterize the subcellular localization of endogenous Axin, we examined confocal sections of larval imaginal disc epithelia stained with an Axin antibody at subapical (Fig 1A–1C) and basolateral (Fig 1D–1I) levels. We confirmed the specificity of the Axin antibody by comparing the Axin signal in wild-type cells with that in juxtaposed mitotic clones of *Axin* null mutant cells (labeled -/- in panel). Double labeling with Armadillo/β-catenin antibody provided a reference for subcellular localization. As expected, Armadillo was enriched at the adherens junctions that demarcate the subapical plasma membrane [27] (Fig 1C) and was also observed in the cytoplasm of *Axin* null mutant cells, resulting from inactivation of the destruction complex [28] (Fig 1A and 1C). Weak background staining was observed in the *Axin* null mutant clones; however the Axin antibody revealed strong and uniform endogenous Axin signal in the apical cytoplasm of wild-type cells, at and above the level of the adherens junctions (Fig 1B).

In contrast with its uniform localization in apical sections, Axin signal was prominent at the cell cortex in basolateral sections, and in particular at the vertices between neighboring cells (Fig 1D–1F). The Axin signal was markedly reduced in *Axin* null mutant cells, verifying its specificity. To determine the localization of cortical Axin with respect to the basolateral cell membrane, we co-stained imaginal discs with antibodies against Axin and Fas III, which demarcates the basolateral cell membrane [29]. Axin staining partially overlapped that of Fas III, but was not identical (Fig 1G–1I): close examination revealed Axin in puncta at or juxtaposed with the basolateral plasma membrane (Fig 1G’–1I’), even at regions far from the Wingless-expressing cells. To determine whether this unanticipated Axin localization was restricted to larval stages, we also co-stained pupal wings, in which Wingless expression in very restricted [30], with antibodies against Axin and Discs Large (Dlg), another basolateral membrane marker [31]. Importantly, by comparison with cells in the larval wing disc, the larger size of cells in pupal wings permitted unequivocal discrimination between cell membrane and cytoplasm. As observed in larval wing discs, Axin was present ubiquitously in puncta juxtaposed with the basolateral membrane of epithelial cells in pupal wings (Fig 1J–1L). These findings revealed that in addition to a diffusely distributed cytosolic pool, Axin is localized in membrane-proximal puncta throughout the wing epithelium at multiple developmental stages in the absence of Wingless stimulation, indicating that the localization of Axin to membrane-associated puncta occurs independently of Wingless exposure.

To distinguish the cytoplasmic and membrane-associated Axin pools using an independent approach, we fractionated lysates from cultured Drosophila embryonic S2R+ cells and analyzed these lysates by immunoblotting with Axin antibody. The transmembrane protein LRP6/Arrow and the cytosolic protein α-tubulin were used as controls for the efficiency of subcellular fractionation (Fig 1M). Consistent with our *in vivo* observations, endogenous Axin was present not only in the cytosolic fraction, but also the membrane fraction, even in the absence of Wingless stimulation (Fig 1M). Quantification indicated that endogenous Axin is present almost equally in the cytoplasmic and membrane fractions (Fig 1N). Notably, cytoplasmic and membrane-associated Axin displayed different migration rates in SDS-PAGE, suggesting that Axin from each pool is subject to differential post-translational modification in these cells (Fig 1M).

To further investigate the subcellular distribution of endogenous Axin in the unstimulated state, we fractionated lysates from wild-type Drosophila embryos that were collected within 2.5 hours of development, which is prior to the onset of Wingless expression [32]. Immunoblots with Axin antibody revealed that Axin was present nearly equally in the cytosolic and membrane fractions (Fig 1O and 1P). Although we cannot exclude the possibility that some Axin is present in cytoplasmic vesicles, these results, coupled with our immunostaining data from larval and pupal wings, support a model in which a pool of Axin is juxtaposed with the membrane in the unstimulated state.

### Apc promotes localization of Axin to puncta juxtaposed with the cell membrane in the unstimulated state

We sought to determine if the localization of Axin to membrane-associated puncta occurs through interaction with its binding partners. We first examined cells that were devoid of the co-receptors required for the response to Wingless stimulation: LRP6/Arrow and the functionally redundant Frizzled (Fz) and Frizzled 2 (Dfz2) [33–36]. In clones of *arrow* null mutant cells in the larval wing discs, the localization of Axin near the basolateral plasma membrane was the same as in neighboring wild-type cells (S1A–S1C Fig). Similarly, in clones of *fz Dfz2* double null mutant cells, the localization of Axin was indistinguishable from that of the neighboring wild-type cells (S1D–S1F Fig). These findings suggested that Axin localization to peripheral puncta does not require interaction with Frizzled or Arrow, and provided further evidence that the existence of a membrane-associated Axin pool does not require Wingless pathway activation nor the interaction of Wingless with Arrow or Frizzled.

**Fig 2:**
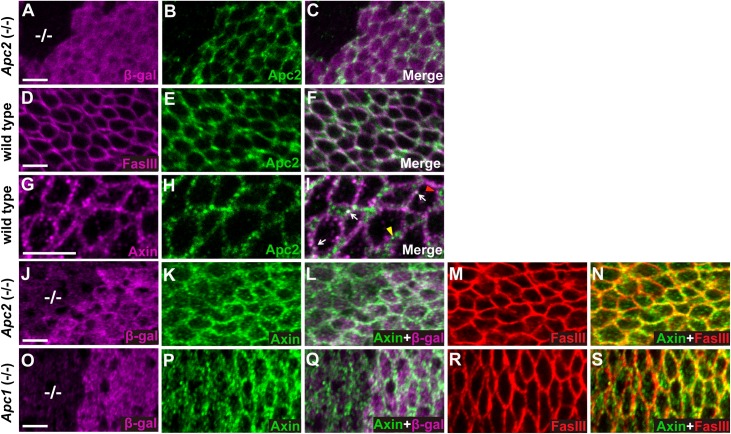
Apc is required for the localization of Axin to puncta juxtaposed with cell membrane. Images of third instar larval wing imaginal discs stained with indicated antibodies; genotypes at left margin. (A-C) Apc2 antibody revealed specific staining of endogenous Apc2 (green), which is absent in *Apc2*^*19*.*3*^ null mutant clones (marked by the absence of β-gal (magenta), -/- in A). (D-F) Double staining with Fas III (magenta) and Apc2 antibodies indicated that Apc2 partially overlaps Fas III and is enriched at cell cortex. (G-I) Double staining with Apc2 and Axin (magenta) antibodies reveals that Apc2 is present at some membrane-proximal Axin puncta (white arrow), whereas distinct Apc2 or Axin puncta are also observed (yellow and red arrowheads respectively). (J-N) Wing disc triple labeled with β-gal (J, magenta), Axin (K, green) and Fas III (M, red) antibodies. Merge of Axin and β-gal is shown in (L), and merge of Axin and Fas III is in (N). *Apc2*^*19*.*3*^ homozygous null mutant clones are marked by the absence of β-gal (-/- in J). Endogenous Axin staining indicates reduced Axin puncta at the basolateral membrane in *Apc2*^*19*.*3*^ null mutant clones (K, L, N). Fas III localization is not disrupted in *Apc2*^*19*.*3*^ mutant clones (M). (O-S) Wing discs bearing *Apc1*^*Q8*^ null mutant clones (marked by the absence of β-gal staining, -/- in O) were stained with β-gal (O), Axin (P) and Fas III (R) antibodies. Merge of Axin and β-gal is shown in (Q), and merge of Axin and Fas III is in (S). Axin puncta are reduced at the basolateral membrane in *Apc1*^*Q8*^ mutant clones (P, Q, S). Fas III localization is not disrupted in *Apc1*^*Q8*^ mutant clones (R). Scale bar: 5μm.

Apc2, which binds Axin in the destruction complex, is present both in the cytoplasm and at the cell cortex in Drosophila embryos [37, 38]. To determine the subcellular localization of Apc2 in larval wing imaginal discs, we examined endogenous Apc2 using an Apc2 antibody. Immunostaining revealed strong signal in wild-type tissue that was markedly reduced in juxtaposed clones of *Apc2* null mutant cells, verifying the specificity of the Apc2 antibody (Fig 2A–2C). Double staining with Fas III antibody revealed that at the level of the basolateral cell membrane, Apc2 was present not only in the cytoplasm, but also enriched at the cell cortex (Fig 2E). The Apc2 signal overlapped, but was distinct from Fas III and was prominent at the vertices between neighboring cells (Fig 2D–2F). Double labeling experiments revealed that Apc2 and Axin co-localized at some puncta that were juxtaposed with cell membrane (Fig 2G–2I), suggesting that these puncta may represent sites of the destruction complex.

As Axin partially co-localizes with Apc2, we sought to determine if Apc promotes Axin localization in puncta juxtaposed with the cell membrane. We examined Axin staining in either *Apc2* or *Apc1* null mutant clones in larval wing imaginal discs. In sharp contrast to its punctate staining at the periphery of wild-type cells, Axin staining was more diffuse in *Apc2* or *Apc1* mutant clones, although some Axin puncta remained (Fig 2J–2N and 2O–2S). Fas III staining confirmed that the morphology of cells in *Apc2* or *Apc1* clones was the same as the neighboring wild-type cells (Fig 2M and 2R), indicating that the decreased Axin localization to puncta at the cell periphery was not a secondary consequence of disrupted cell morphology. Importantly, previous genetic and biochemical studies had demonstrated that endogenous Axin is not destabilized as a consequence of Apc loss [16]; therefore, the decreased localization of Axin in peripheral puncta is not secondary to decreased Axin levels in *Apc* mutant cells. Based on these results, we conclude that Apc promotes Axin localization to puncta at the cell periphery in the unstimulated state.

### An *in vivo* system for analysis of membrane-associated Axin

**Fig 3:**
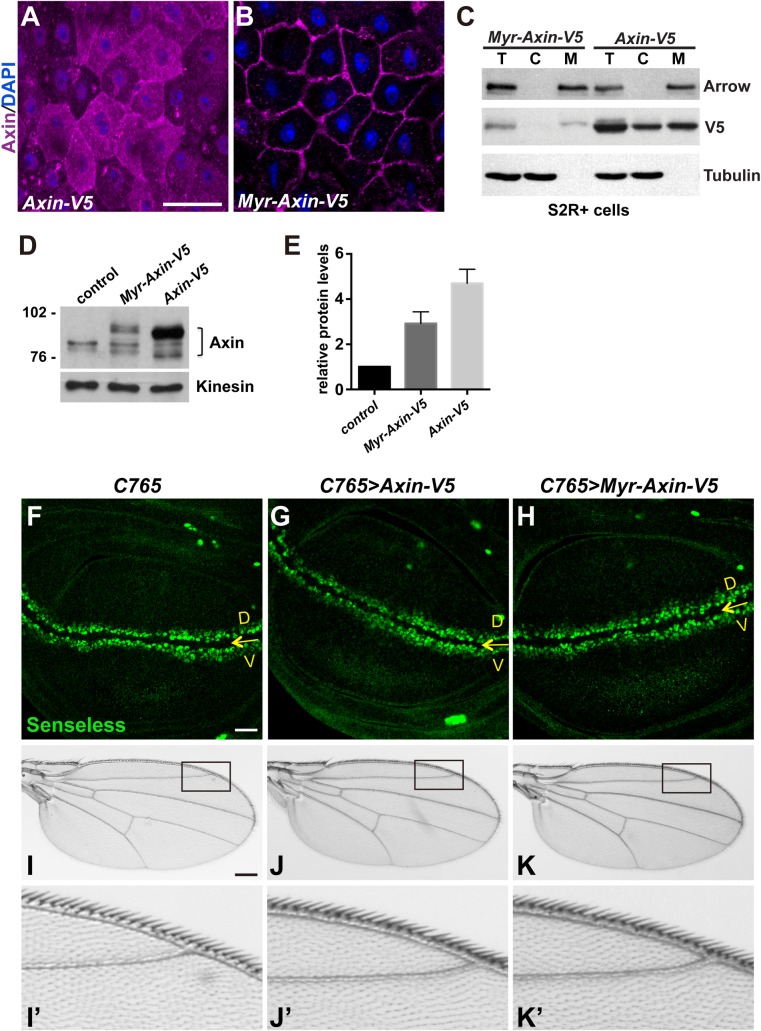
An *in vivo* system for analysis of membrane-associated Axin (A-B) Confocal images of adult midguts expressing *Axin-V5* or *Myr-Axin-V5* using the *Myo1A-Gal4* driver. Midguts were stained with anti-Axin (magenta) and DAPI (blue). (C) Subcellular fractionation of lysates from S2R+ cells transfected with indicated plasmids. The total cell lysate (T), cytoplasmic (C) and membrane (M) fractions were analyzed by immunoblotting with V5 antibody. Myr-Axin-V5 is present mainly in the membrane fraction, whereas Axin-V5 is present in both the cytoplasmic and membrane fractions. The efficiency of the fractionation was assayed by the presence of Arrow and Tubulin, membrane and cytoplasmic markers, respectively. (D) Total Axin levels in wing imaginal discs expressing indicated transgenes with the *C765-Gal4* driver. *C765-Gal4* flies were used as control. (E) Quantification of the relative total Axin protein levels in wing discs with indicated genotypes. Results were obtained from four independent experiments with a representative blot shown in (D). Values indicate mean ± SD. (F-K) Expressing *Axin-V5* or *Myr-Axin-V5* with the *C765-Gal4* driver in larval wing discs does not disrupt expression of the Wingless target gene *senseless* (F-H), or the morphology of adult wings (I-K). Yellow arrows in (F-H) indicate the dorsoventral boundary of the larval wing disc. Boxed areas in (I-K) are shown in (I’-K’). 15–20 flies of each genotype were examined. Scale bar: 20μm.

Next, we sought to analyze the function and regulation of membrane-associated Axin. To address this, we generated an *Axin* transgene (*Myr-Axin-V5*) in which we added to the Axin amino-terminus a ten amino acid myristoylation sequence from the Drosophila Src protein [39], which is known to target proteins to membranes [40, 41]. *Myr-Axin-V5* and an *Axin-V5* transgene that does not contain the myristoylation sequence but is otherwise identical [18], were integrated at the same genomic site to allow for their direct comparison in the absence of transcriptional position effects. To investigate the effect of the myristoylation sequence on the subcellular localization of Axin, we analyzed the adult midgut, in which the large size of absorptive epithelial cells (enterocytes) permits unequivocal distinction between plasma membrane and cytoplasm. In contrast with Axin-V5, which localized to both cytoplasm and cell membrane, Myr-Axin-V5 was localized predominantly at the cell membrane (Fig 3A and 3B), indicating that the myristoylation sequence was effective in targeting Axin to the plasma membrane, consistent with previous work [42]. To test this conclusion using an independent approach, we expressed Myr-Axin-V5 or Axin-V5 in S2R+ cells and determined their distribution using subcellular fractionation. Axin-V5 was distributed nearly equally in the cytoplasmic and membrane fractions; in contrast, Myr-Axin-V5 was highly enriched in the membrane fraction (Fig 3C). We conclude that the myristoylation sequence is sufficient for the membrane targeting of Axin.

We previously established a system to express an *Axin-V5* transgene within the Axin physiological threshold in larval wing imaginal discs using the *C765-Gal4* or *71B-Gal4* drivers [16]. We sought to use this system to express membrane-targeted Axin within physiological range and to study its regulation. Therefore, we first examined Axin protein levels in lysates from control wing discs or those expressing either the *Myr-Axin-V5* or *Axin-V5* transgene with the *C765-Gal4* driver (Fig 3D). Quantification revealed a two-fold and four-fold increase in Axin levels in wing discs expressing *Myr-Axin-V5* or *Axin-V5* respectively, by comparison with controls expressing only endogenous Axin (Fig 3E). These levels of Myr-Axin-V5 and Axin-V5 are below the threshold at which Axin overexpression disrupts Wingless signaling in wing discs [16]. To test this conclusion using an independent approach, we determined whether Myr-Axin-V5 expression at these levels disrupted Wingless signaling in wing discs. Staining with an antibody against Senseless, a Wingless signaling readout at the dorso-ventral boundary of the third instar larval wing imaginal disc, revealed a pattern that was indistinguishable from controls (Fig 3F–3H). In addition, expression of Axin-V5 or Myr-Axin-V5 using the *C765-Gal4* driver did not disrupt the morphology of adult wings (Fig 3I–3K and 3I’–3K’). Similar results were obtained when Axin-V5 or Myr-Axin-V5 was expressed using the *71B-Gal4* driver (S2 Fig). Taken together, these findings indicated that when expressed within physiological range, membrane-tethered Axin does not disrupt normal Wingless signaling.

### Membrane-associated Axin is sufficient for Wingless signaling

**Fig 4:**
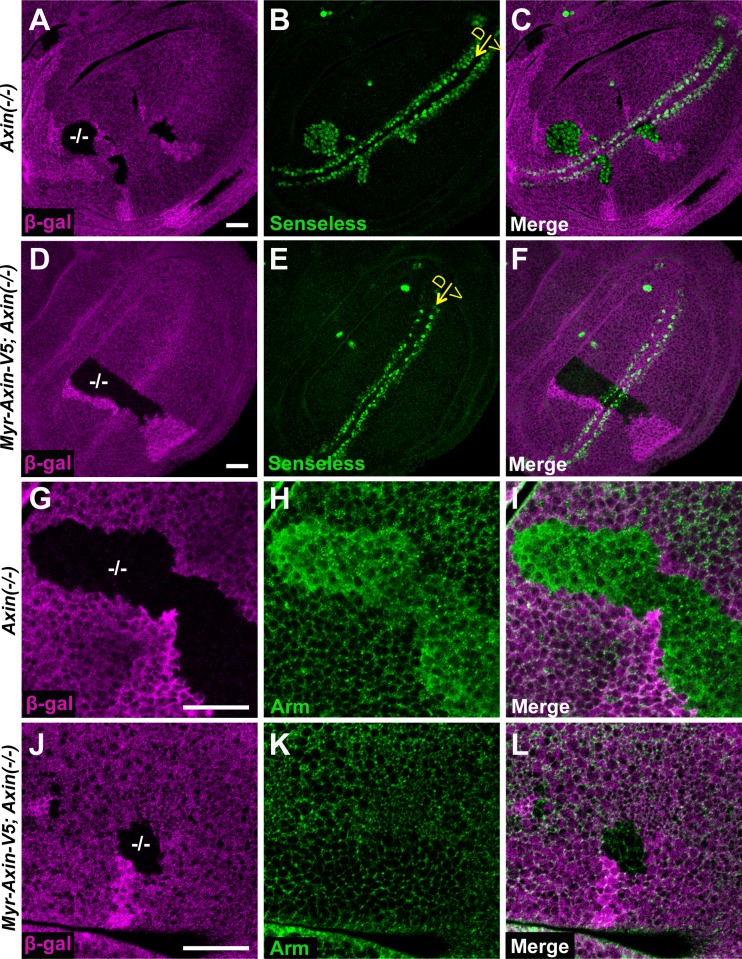
Membrane-associated Axin is sufficient for Wingless signaling (A-C) Confocal images of third instar larval wing discs with *Axin*^*s044230*^ null mutant clones (marked by the absence of β-gal in A (magenta)). The Wingless target gene *senseless* (green) is ectopically activated in *Axin* mutant clones (B and C). (D-F) Expressing *Myr-Axin-V5* in wing discs using the *71B-Gal4* driver restores normal *senseless* expression in *Axin* null mutant clones. Yellow arrows indicate the dorsoventral boundary. (G-I) Arm is ectopically stabilized in *Axin* null mutant clones (marked by the absence of β-gal in G). (J-L) Expressing *Myr-Axin-V5* in wing discs using the *71B-Gal4* driver suppresses aberrant Arm stabilization in *Axin* null mutant clones. Scale bar: 20μm.

To determine whether membrane-targeted Axin expressed within physiological range was sufficient to replace the function of endogenous Axin, we tested whether Myr-Axin-V5 could restore normal signaling in *Axin* null mutants. As expected, *senseless* was ectopically expressed in *Axin* null mutant clones, resulting from the aberrant activation of Wingless signaling (Fig 4A–4C). In contrast, expression of Myr-Axin-V5 in wing discs using the *71B-Gal4* driver fully prevented ectopic *senseless* expression even in the absence of endogenous Axin, and importantly, did not disrupt physiological *senseless* expression (Fig 4D–4F). Similarly, the aberrant stabilization of cytoplasmic Armadillo in *Axin* mutant cells (Fig 4G–4I) was fully prevented in cells expressing Myr-Axin-V5 (Fig 4J–4L). These results suggested that membrane-bound Axin can functionally replace endogenous Axin in both the destruction complex and signalosome. Our results in wing imaginal discs are consistent with the ability of membrane-bound Axin to functionally replace endogenous Axin during embryogenesis [42]. Taken together, these findings suggested that membrane-associated Axin is sufficient for proper regulation of Wingless signaling.

### Membrane association promotes Axin degradation through Tnks-dependent ADP-ribosylation

**Fig 5:**
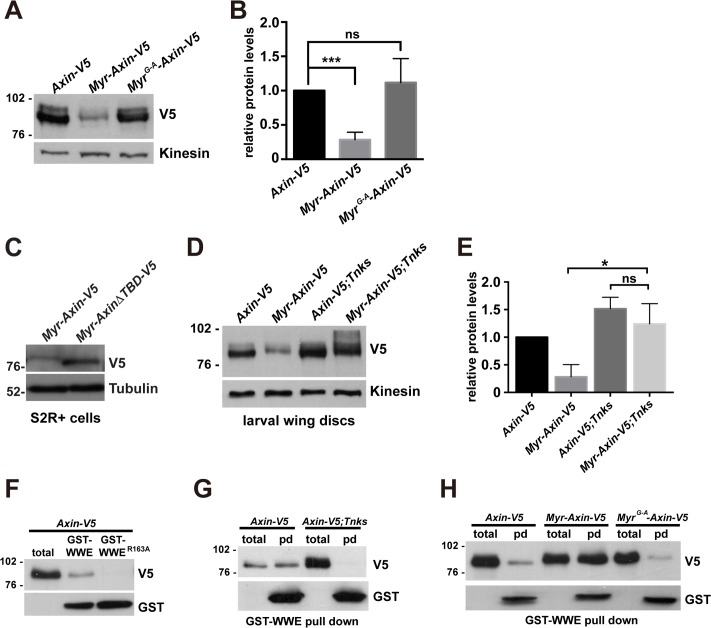
Membrane-association promotes Axin degradation through Tankyrase-dependent ADP-ribosylation (A) Lysates from third instar larvae expressing indicated transgenes with the *C765-Gal4* driver were analyzed by immunoblotting. Myr-Axin-V5 is present at much lower levels than Axin-V5 or Myr^G-A^-Axin-V5. Kinesin was used as a loading control. (B) Quantification of the relative levels of indicated proteins. Results were obtained from four independent experiments with representative blot shown in (A). Values indicate mean ± SD. *** *p*<0.001, ns: not significant (t-test). (C) S2R+ cells were transfected with the indicated plasmids. Lysates were analyzed by SDS-PAGE. Immunoblotting with V5 antibody revealed that Myr-AxinΔTBD-V5 is present at higher levels than Myr-Axin-V5. Tubulin was used as a loading control. (D) Lysates from third instar larvae of indicated genotypes were analyzed by immunoblotting. Transgene was expressed with the *C765-Gal4* driver. Eliminating Tnks restores the protein levels of Myr-Axin-V5. Kinesin was used as a loading control. (E) Quantification of the relative protein levels of Axin-V5 or Myr-Axin-V5 from lysates of third instar larvae of indicated genotype. Results were obtained from three independent experiments with a representative blot shown in (D). Values indicate mean ± SD. * p = 0.0163, ns: not significant (t-test). (F) Use of GST-WWE pull-down assay for detection of ADP-ribosylated Axin. Lysates from third instar larvae expressing *Axin-V5* with the *C765-Gal4* driver were incubated with GST-WWE or GST-WWE^R163A^ beads. Axin-V5 is pulled down by GST-WWE, but not GST-WWE^R163A^, suggesting this assay specifically detects ADP-ribosylated Axin. (G) GST-WWE pulldown from lysates of third instar larvae expressing indicated transgene with the *C765-Gal4* driver. ADP-ribosylation of Axin is abolished in *Tnks*^*19*^ null mutants. Total indicates total larval lysates and pd indicates samples pulled down with GST-WWE beads. (H) GST-WWE pulldown from lysates of third instar larvae of indicated genotypes. Myr-Axin-V5 is highly ADP-ribosylated by comparison with Axin-V5 or Myr^G-A^-Axin-V5.

We observed, unexpectedly, that levels of Myr-Axin-V5 were lower than those of Axin-V5, as revealed by immunoblotting of lysates from cultured cells and larval wing discs expressing these respective proteins (Fig 3C, lane 1 and lane 4 and Fig 3D and 3E), suggesting that the membrane targeting of Axin may result in its destabilization. To rule out the possibility that the addition of amino acids to the Axin amino-terminus inadvertently disrupted Axin regulation, we generated a control transgene, *Myr*^*G-A*^*-Axin-V5*, in which substitution of a single amino acid (Gly to Ala) within the myristoylation sequence is known to abolish myristoylation and membrane localization [43]. We found that Myr^G-A^-Axin-V5 was present at much higher levels than Myr-Axin-V5 in larval wing discs (Fig 5A and 5B). The same result was observed in lysates from adult midguts (S3A Fig), suggesting that the decreased levels of Myr-Axin-V5 were a specific consequence of its membrane association. Together, these findings suggested that the association of Axin with the membrane promotes its proteolysis.

As we had found that targeting Axin to the membrane results in its destabilization, we sought to determine whether Tnks, which is known to target Axin for degradation, promotes the proteolysis of membrane-associated Axin. To address this question, we generated a transgene encoding membrane-tethered Axin lacking the Tnks binding domain (*Myr-AxinΔTBD-V5*). We postulated that if Tnks were important for Axin degradation at the membrane, then Myr-AxinΔTBD-V5 would be more stable than Myr-Axin-V5. Expression of *Myr-Axin-V5* or *Myr-AxinΔTBD-V5* in S2R+ cells supported this hypothesis; Myr-AxinΔTBD-V5 was present at higher levels than Myr-Axin-V5 (Fig 5C). These results suggested that Axin degradation at the membrane required the Tnks binding domain of Axin.

To test whether Tnks promotes the degradation of membrane-associated Axin *in vivo*, we expressed *Axin-V5* or *Myr-Axin-V5* using the *C765-Gal4* driver in wing discs of either wild-type or *Tnks* mutants and compared Axin levels. We found that inactivation of Tnks significantly increased the levels of Myr-Axin-V5 to levels comparable to Axin-V5 (Fig 5D and 5E), suggesting that Tnks-dependent proteolysis is a major mechanism that promotes the degradation of membrane-associated Axin. Supporting this conclusion, elimination of Tnks also increased the levels of Myr-Axin-V5 in adult midguts (S3B Fig). Furthermore, immunostaining revealed that even at these increased levels, Myr-Axin-V5 still localized predominately to the cell membrane of adult midgut enterocytes, suggesting that the subcellular distribution of Myr-Axin-V5 is not due to its lower levels (S3C–S3E Fig). Together, these findings provided evidence that Tnks-dependent Axin proteolysis destabilizes Axin at the membrane.

As Tnks is known to target Axin for proteolysis through ADP-ribosylation, we sought to determine if association with the membrane promotes Axin ADP-ribosylation, and thereby Axin degradation. To detect the low levels of ADP-ribosylated Axin, we took advantage of the finding that ADP-ribosylated Axin is recognized by the RING-type E3 ubiquitin ligase RNF146/Iduna for ubiquitination and subsequent degradation [13, 14, 44, 45]. The WWE domain of RNF146 interacts directly with poly(ADP-ribose) in Axin to promote its ubiquitination. Pull downs using the WWE domain of RNF146 coupled to glutathione S-transferase (GST) specifically detect ADP-ribosylated Axin [14]. Therefore, to determine the level of ADP-ribosylated Axin *in vivo*, lysates from larvae expressing *Axin-V5* were subjected to pull down with GST-WWE or the GST-WWE^R163A^ control, in which an arginine to alanine substitution abolishes interaction with poly(ADP-ribose) [14]. The pull down of Axin-V5 with GST-WWE, but not the GST-WWE^R163A^ control, confirmed the specificity of the assay (Fig 5F). We further confirmed the specificity of the pull-down experiments through Tnks inactivation: ADP-ribosylated Axin-V5 was not detected in the GST-WWE pull-downs in lysates from *Tnks* null mutant larvae, in contrast with wild-type (Fig 5G).

To determine the extent to which the membrane-associated pool of Axin is ADP-ribosylated *in vivo*, we expressed *Axin-V5*, *Myr-Axin-V5* or *Myr*^*G-A*^*-Axin-V5* in the wing discs of third instar larvae. As myristoylation of Axin-V5 results in markedly reduced levels, more larvae expressing *Myr-Axin-V5* were used to obtain a comparable input for the GST-WWE pull-down assay. We found that the level of ADP-ribosylation of Myr-Axin-V5 was much higher than that of Axin-V5 or Myr^G-A^-Axin-V5 (Fig 5H). These findings suggested that membrane association of Axin results in an increased pool of ADP-ribosylated Axin.

### Axin membrane association does not require Tnks-dependent ADP-ribosylation

**Fig 6:**
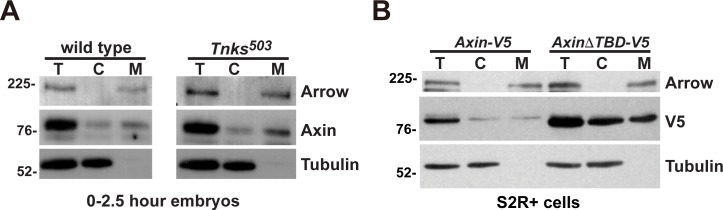
Axin membrane association does not require Tnks-dependent ADP-ribosylation (A) Subcellular fractionation of lysates from 0–2.5 hour old wild-type embryos and *Tnks*^*503*^ null mutant embryos. The total lysates (T), cytoplasmic (C), and membrane (M) fractions were analyzed by SDS-PAGE. Immunoblotting with Axin antibody revealed that Axin is present in both the membrane and cytoplasmic fractions in both wild-type embryos and *Tnks*^*503*^ null mutants. The efficiency of the fractionation was assayed by the presence of Arrow and Tubulin, membrane and cytoplasmic markers, respectively. (B) Subcellular fractionation of lysates from S2R+ cells transfected with the indicated plasmids. The total lysates (T), cytoplasmic (C), and membrane (M) fractions were analyzed by SDS-PAGE. Immunoblot with V5 antibody revealed that Axin-V5 and AxinΔTBD-V5 are localized in both the membrane and cytoplasmic compartments. The efficiency of the fractionation was assayed by the presence of Arrow and Tubulin, membrane and cytoplasmic markers respectively.

As we had found that membrane association destabilizes Axin under basal conditions and that Tnks promotes the degradation of membrane-associated Axin (Figs 5 and S3), we sought to determine whether Tnks-mediated ADP-ribosylation is required for Axin membrane association. To test this hypothesis *in vivo*, we performed subcellular fractionation of lysates from wild-type and *Tnks* null mutant embryos within 2.5 hours of development (prior to Wingless expression) and determined the distribution of endogenous Axin in membrane and cytoplasmic fractions using immunoblots. We found that the subcellular distribution of Axin in *Tnks* null mutant embryos was similar to that in wild-type embryos, with pools of Axin in both the cytoplasmic and membrane fractions (Fig 6A). This finding suggested that Tnks-mediated ADP-ribosylation is not essential for Axin membrane association. To test this conclusion using another approach, we investigated whether the Tnks binding domain of Axin is important for its membrane localization. We fractionated lysates from S2R+ cells expressing *Axin-V5* or *AxinΔTBD-V5*, and examined the distribution of Axin in immunoblots with V5 antibody. Equivalent levels of both Axin-V5 and AxinΔTBD-V5 were present in cytoplasmic and membrane compartments (Fig 6B). These findings indicated that Tnks and the Tnks binding domain of Axin were dispensable for Axin membrane association. We conclude that ADP-ribosylation is not required for Axin membrane association, but that the membrane association of Axin results in increased ADP-ribosylation.

### Wingless stimulation results initially in increased levels of both membrane-associated and cytosolic Axin prior to Axin proteolysis hours later

**Fig 7:**
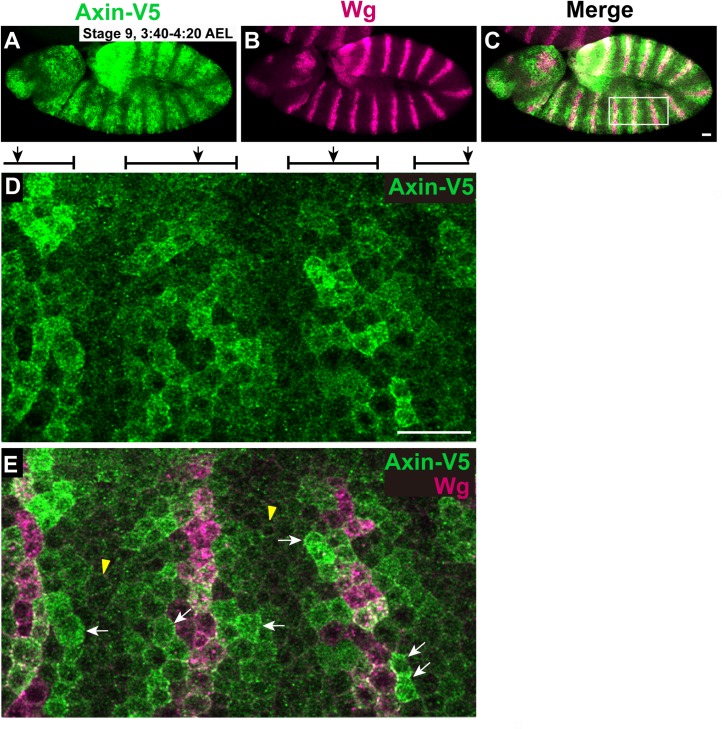
Axin levels are increased in the cytoplasm and at the plasma membrane following Wingless exposure. Confocal images of embryos expressing *Axin-V5* driven by the *mat-Gal4* driver stained with V5 (green) and Wingless (Wg, magenta) antibodies. Anterior left, dorsal up. (A-C) By 30 minutes after the onset of Wg expression (stage 9), Axin-V5 increases in segmental stripes both at the plasma membrane and in the cytoplasm. (D and E) Higher magnification of boxed region in panel (C). Axin-V5 is distributed in wide segmental stripes (brackets on top) that overlap the Wg stripes (black arrows on top). Cells surrounding the Wg stripes display increased Axin intensity that is present at both the cell membrane and within the cytoplasm (white arrows) compared with cells between neighboring Axin stripes (yellow arrowheads). Axin is present in puncta at the cell periphery even in the absence of Wingless (yellow arrowheads). Scale bars: 25 μm.

Previous work based on Axin overexpression suggested that Wingless stimulation induced the bulk relocation of Axin from cytoplasm to cell membrane in Drosophila embryos [21, 22]. Recently, we re-examined the effects of Wingless exposure in Drosophila embryos using an *Axin-V5* transgene that is expressed within two-fold of endogenous levels, is able to replace the function of endogenous Axin, and does not disrupt Wnt signaling [18]. We discovered that Axin levels increase in Wingless-responding cells within thirty minutes of Wingless stimulation, resulting in a segmentally striped pattern of Axin in Drosophila embryos (Fig 7A–7C), prior to Axin degradation hours later, and that this process is conserved in vertebrate cells [18]. We sought to use this *in vivo* system to examine the subcellular localization of Axin. Within the first three hours of embryogenesis, prior to the onset of Wingless expression, Axin was present both in the cytoplasm and at the plasma membrane, as revealed by its partial overlap with the transmembrane protein Neurotactin [46] (S4 Fig). Notably, within thirty minutes following Wingless exposure, Axin puncta were observed near the cell periphery both in cells responding to Wingless stimulation and in those not exposed to Wingless (Fig 7D and 7E). Importantly, we did not detect bulk redistribution of Axin from cytoplasm to membrane in Wingless responding cells, but instead found increased Axin signal in both the cytoplasm and at the plasma membrane (Figs 7D, 7E and S5). These results are in sharp contrast with a previous finding in which overexpressed Axin-GFP associated with the plasma membrane only in cells responding to Wingless stimulation [21]. We conclude that Wingless exposure induces an increase in Axin levels in both the cytoplasm and at the plasma membrane, and not bulk redistribution from cytoplasmic to membrane pools.

**Fig 8:**
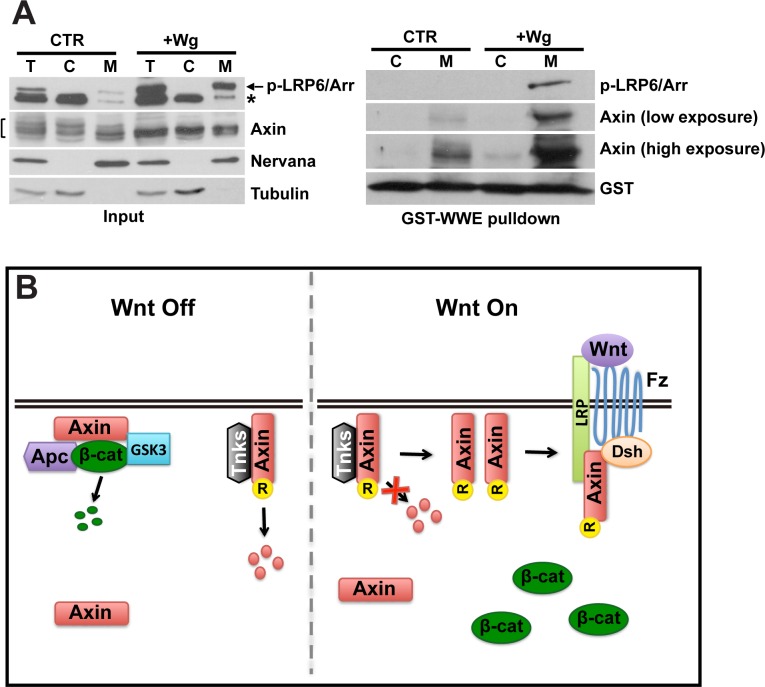
Wingless stimulation results in increased membrane-associated ADP-ribosylated Axin (A, left panel) Subcellular fractionation of lysates from S2R+ cells treated with either control medium (CTR) or Wingless conditioned medium (Wg CM) for 1 hour. The total cell lysates (T), cytoplasmic (C), and membrane (M) fractions were analyzed by SDS-PAGE. Immunoblotting with phosphorylated LRP6 antibody shows activation of the Wnt pathway following treatment with Wg CM. Immunoblotting with Axin antibody shows no significant shift from the cytoplasmic fraction to membrane fraction. Tubulin, a cytoplasmic marker, and Nervana, a membrane marker, were used to assay the efficiency of fractionation. Asterisk indicates a non-specific band with p-LRP6 antibody. (A, right panel) Following cell fractionation, GST-WWE pulldown was performed with cytoplasmic (C) and membrane (M) fractions. Immunoblotting with Axin antibody reveals that the majority of ADP-ribosylated Axin is in the membrane fraction, with little ADP-ribosylated Axin detected in the cytoplasm. Phospho-LRP6 is pulled down by GST-WWE in the membrane fraction in response to Wingless. (B) Model for dual roles of membrane-associated Axin. In the absence of Wnt stimulation, a pool of Axin is localized in membrane-proximal puncta, which might represent the sites of the destruction complex. Membrane-associated Axin is targeted for degradation by Tnks in the unstimulated state, which contributes to maintaining Axin at the limiting concentrations important for regulation of Wnt signaling. Wnt stimulation induces a rapid increase in the level of Axin, and in particular the membrane-associated ADP-ribosylated Axin pool. ADP-ribosylation enhances the interaction of Axin with phospho-LRP6/Arrow, and thus promotes the activation of signaling. R: ADP-ribosylation.

Previous findings demonstrated that Axin levels increase in the membrane fraction following Wnt stimulation [3]. However, accompanying changes in the overall and cytosolic levels of Axin were not documented in that study, and thus it remained possible that the increase of Axin in the membrane fraction occurred concomitantly with an increase in overall Axin levels. To test this possibility, we used an approach that capitalized on our ability to detect endogenous Axin in lysates from cultured Drosophila embryonic cells. We treated S2R+ cells with Wingless conditioned medium (Wg CM), and then subjected the lysates to subcellular fractionation and immunoblotting. Confirming robust Wingless pathway activation, the level of phosphorylated LRP6/Arrow increased rapidly following treatment with Wg CM (Fig 8A, left panel). As reported previously, the total levels of Axin as determined by measuring all Axin isoforms increased rapidly though modestly following Wingless stimulation [18] (Fig 8A, left panel, lane 1 and 4). Furthermore, several slower migrating bands detected by the Axin antibody in the unstimulated state were not present after Wingless stimulation, suggesting an alteration in post-translational modification in response to pathway activation. This result is consistent with previous reports that mammalian Axin is dephosphorylated following Wingless exposure [5, 24, 25, 47]. Importantly, supporting our immunostaining results in fly embryos, the relative ratio of Axin in the cytoplasmic and membrane fractions was similar in the absence or presence of Wingless stimulation, indicating that bulk redistribution of Axin from cytoplasm to membrane in response to Wingless does not occur (Fig 8A, left panel).

Our previous work revealed that after Wnt stimulation, the level of ADP-ribosylated Axin increases, and ADP-ribosylation promotes the interaction of Axin with phospho-LPR6/Arrow in both Drosophila and human cells [18]. To further investigate whether the cytosolic and membrane pools of Axin are differentially regulated in response to Wingless stimulation, we examined the subcellular localization of ADP-ribosylated Axin by subjecting cytosolic and membrane fractions from S2R+ cell lysates to the GST-WWE pull-down assay. Consistent with our hypothesis that Axin is ADP-ribosylated primarily at the membrane, we found that the vast majority of ADP-ribosylated Axin is present in the membrane fraction in the unstimulated state (Fig 8A, right panel, lane 1 and 2). We next determined whether Wingless exposure alters the subcellular localization of ADP-ribosylated Axin. We confirmed that Wingless stimulation induces a marked increase in the level of ADP-ribosylated Axin, and furthermore, found that this Wingless-induced increase in ADP-ribosylated Axin is confined largely to the membrane (Fig 8A, right panel). Consistent with our previous finding that Axin ADP-ribosylation enhances its interaction with phospho-LRP6 [18], phospho-LRP6 was also pulled down by GST-WWE in the membrane fraction following Wingless stimulation, likely through the interaction with ADP-ribosylated Axin (Fig 8A, right panel). We conclude that Wingless exposure does not result in bulk redistribution of Axin from the cytosol to the plasma membrane, but rather induces increased levels of both cytoplasmic and membrane-associated Axin, with a preferential increase in the level of ADP-ribosylated Axin at the membrane, which likely facilitates the association between Axin and phospho-LRP6 and thus the activation of Wingless pathway.

## Discussion

Although Axin is a core component of Wnt pathway, the regulation of Axin remains poorly understood, largely due to technical challenges in studying endogenous Axin, which is present at low levels. Here, we capitalized on the sensitivity of an antibody that detects endogenous Drosophila Axin *in vivo* and in cultured cells to study Axin regulation. We found that even in the absence of Wnt stimulation, a pool of Axin is present in puncta that are localized at or proximal to the basolateral cell membrane *in vivo*. Subcellular fractionation of Drosophila embryos and cultured embryonic cells supported the membrane association of Axin in unstimulated conditions. These results were further supported by an experimental system in Drosophila embryos in which exogenous Axin was expressed at levels that are within two-fold of endogenous Axin and thus does not disrupt Wnt signaling [18]. We utilized this system to re-examine the proposed redistribution of Axin from cytosol to plasma membrane following Wingless stimulation. In contrast with previous work based on Axin overexpression at levels that abrogated Wingless signaling [21], we found that the initial response to Wingless stimulation is not bulk translocation of Axin from cytosol to plasma membrane, but instead a modest increase in both cytoplasmic and membrane-associated Axin, and is followed by Axin proteolysis. Furthermore, evidence from our subcellular fractionation studies of Drosophila S2R+ cells responding to Wingless exposure also did not support the bulk relocation of Axin from cytoplasm to cell membrane in response to stimulation, but did suggest changes in Axin post-translational modification (see below).

Previous work indicated that Axin is sequestered in multivesicular bodies (MVBs) that form following Wnt stimulation [48]; however a subsequent study revealed that MVB formation is not required for Wnt pathway activation in Drosophila [49]. The membrane-proximal Axin puncta we have observed are unlikely to represent MVBs, since these puncta are found in the absence of Wnt exposure; however, the presence of these puncta may suggest that Axin associates with other types of vesicles juxtaposed with the plasma membrane. Previously, endogenous Axin was observed in puncta that were termed “degradasomes” in mammalian cultured cell lines; however these puncta were detected only when both Axin and Tnks levels were aberrantly increased by the use of Tnks inhibitors [50, 51]. The presence of Axin in widespread puncta was also reported following overexpression of mammalian or Drosophila Axin and/or Apc, and those puncta were proposed to contain the destruction complex and promote β-catenin degradation [52–56]. Our documentation of membrane-proximal puncta containing Axin at endogenous levels furthers this previous work, and also reveals that the formation of these Axin puncta is largely dependent on the activity of endogenous Apc. As some of these Axin-containing puncta overlap the endogenous Apc signal, they may indeed represent the sites of destruction complex activity. How Apc promotes the localization of Axin to these membrane proximal puncta awaits further investigation, but may involve previously proposed roles for Apc in the facilitation of Axin multimerization [22, 56].

We find that the membrane association of Axin promotes its proteolysis in the unstimulated state, and thus is important to maintain Axin at concentration-limiting levels. Previous studies revealed that Tnks-dependent Axin degradation is one of the mechanisms that maintain Axin at low levels in both mammalian and Drosophila cells [12, 15, 16], and our results herein indicate that Tnks targets the membrane-associated pool of Axin for ADP-ribosylation and degradation. Therefore, we propose that Tnks-mediated ADP-ribosylation of Axin at the membrane is important to control Axin levels under basal conditions. Previous work revealed that mammalian Tnks localizes to the lateral membrane in polarized epithelial cells [57]; therefore, it is possible that enrichment of Tnks activity at the membrane promotes the proteolysis of membrane-associated Axin. Alternatively, membrane association may simply result in a local enrichment as Axin moves from a three-dimensional space (the cytoplasm) to a 2-dimensional surface (the plasma membrane), and could thereby promote Tnks-dependent Axin ADP-ribosylation and subsequent degradation. Whether the cytoplasmic and membrane-associated pools of Axin are targeted for proteolysis through distinct mechanisms awaits further studies.

Whereas ADP-ribosylated Axin is associated primarily with the membrane fraction, the membrane-association of Axin does not require ADP-ribosylation. In addition, three findings support the conclusion that the association of Axin with the membrane does not require Wingless stimulation: membrane-proximal Axin puncta are observed ubiquitously in larval imaginal discs, Axin is enriched at the cell membrane prior to the onset of Wingless expression during embryogenesis, and this membrane association is not disrupted by inactivation of the Wingless co-receptors LRP6/Arrow and Frizzled, both of which are essential for the response to Wingless stimulation. Previous studies in Xenopus embryos revealed that Axin at the plasma membrane could be partially precipitated using concanavalin A beads, indicating that a pool of Axin is associated with membrane glycoproteins [52]. The precise mechanisms that maintain Axin’s membrane association await further investigation.

Taken together, our results indicate that a pool of Axin localizes to puncta at or near the cell membrane and is targeted for degradation by Tnks under basal conditions. We propose that this set up not only maintains Axin at low levels in the absence of Wnt stimulation, but also provides a mechanism that can trigger the rapid transition in Axin activity that facilitates signaling following Wnt stimulation. In support of this hypothesis, our recent work revealed that Axin levels, and in particular the ADP-ribosylated pool of Axin, increase rapidly after Wnt stimulation and that ADP-ribosylation enhances the association of Axin with phospho-LRP6/Arrow [18]. Herein, by analyzing the subcellular distribution of endogenous Axin, we found that the vast majority of ADP-ribosylated Axin is membrane-associated under basal conditions. In response to Wnt exposure, the increased pool of ADP-ribosylated Axin remains membrane-associated, and thus may further enhance the Axin-phospho-LRP6 interaction through both increased local concentration and increased affinity. The mechanisms underlying the rapid increase in Axin levels after Wnt exposure, and specifically in the ADP-ribosylated Axin pool, await further investigation, but we speculate that this response results from the rapid inhibition of Axin proteolysis that follows Wnt exposure. Furthermore, immunoblots with our Axin antibody suggest changes in post-translational modification of endogenous Axin in response to Wnt stimulation, consistent with the known Wnt-induced dephosphorylation of Axin that is thought to diminish the association between Axin and β-catenin [5, 24, 25]. Therefore, we hypothesize that modulation of two post-translational modifications in Axin—ADP-ribosylation as documented herein, and dephosphorylation as documented previously [5, 24]—promote the initial response to Wnt stimulation. Specifically, dephosphorylation of Axin inhibits the destruction complex [24, 47], whereas ADP-ribosylation of the membrane-associated pool of Axin enhances the association of Axin with phospho-LRP6, a step that promotes assembly of the signalosome [18].

Based on these findings, we propose a revised model in which the membrane association of Axin is important for its regulation in both unstimulated and stimulated states (Fig 8B). In the absence of Wnt ligands, membrane-associated Axin is a substrate for ADP-ribosylation and ubiquitination, which targets Axin for degradation and thereby controls its limiting concentration that is important for the regulation of Wnt signaling. We speculate that Wnt stimulation rapidly inhibits Axin degradation, thereby inducing an increase in the level of membrane-associated, ADP-ribosylated Axin. As ADP-ribosylation enhances the interaction of Axin with phospho-LRP6 [18], the Wnt-induced increase in levels of membrane-associated ADP-ribosylated Axin likely promotes the role of Axin in signaling activation. An extracellular gradient of Wingless protein forms on the basolateral surface of epithelial cells [58, 59]; therefore we postulate that in response to Wingless stimulation, Axin associated with or near the basolateral cell membrane jump-starts the rapid association of Axin with LRP6, which is among the earliest responses to Wnt exposure [3].

## Materials and Methods

### Flies and genetics

A complete deletion of the *Axin* gene, *Axin*^*18*^, was isolated by *FLP*-mediated trans-recombination between FRT sites [60] in *PBac{RB}Mgat2*^*e01270*^ and *PBac{WH}Axn*^*f01654*^ (both obtained from the Exelixis collection at Harvard Medical School). Potential deletions were identified by lethal complementation tests with the mutant allele *Axin*^*s044230*^.

Other stocks: *Apc1*^*Q8*^ [61], *Apc2*^*19*.*3*^ [62], *Ubx-FLP* [63], *C765-Gal4* (BDSC) [64], *vestigial-Gal4 UAS-FLP* [35], *FRT82B arm-lacZ* [65] (provided by J. Treisman, Skirball Institute, New York), *cycA*^*C8LR1*^ *ubi-GFP FRT2A* [66], *engrailed-Gal4 UAS-FLP* (provided by E.Piddini, NIMR, London), *fz1*^*P21*^ *Dfz2*^*C2*^ *FRT2A* [35], *FRT42D ubi-GFP PCNA*^*775*^ [67], *hh-Gal4 UAS-FLP* [66], *FRT42D arr*^*2*^ [33], *Myo1A-Gal4* [68], *hsFLP1* [69], *71B-Gal4* (BDSC) [64], *Axin*^*s044230*^ [28], *Tnks*^*19*^ [17], and *Tnks*^*503*^ [17]. The maternal *α4-Gal4*:*VP16* driver (*mat-Gal4*; line 67) contains the maternal *tubulin* promoter from *αTub67C* and the 3' UTR from *αTub84B* [70, 71]. Canton S flies were used as wild-type controls. All crosses were performed at 25°C unless otherwise indicated.

### Generation of somatic mutant mitotic clones

Somatic mitotic mutant clones were generated by *FLP*-mediated recombination [72]. Clones induced by *hsFLP1* were generated by subjecting first and second instar larvae to a 37°C heat shock for 2hr and analyzed at third instar larval stage. Genotypes for generating mutant clones are as follows:

*Axin* mutant clones: *vestigial-Gal4 UAS-FLP/+; FRT82B Axin*^*18*^*/FRT82B arm-lacZ* (Fig 1) or *hsFLP1/+; FRT82B Axin*^*18*^*/FRT82B arm-lacZ* (Fig 4)

*arrow* mutant clones: *FRT42D arr*^*2*^*/FRT42D ubi-GFP PCNA*^*775*^*; hh-Gal4 UAS-FLP/+*

*fz Dfz2* double mutant clones: *en-Gal4 UAS-FLP/+; fz*^*P21*^ *Dfz2*^*C2*^ *FRT2A/cycA*^*C8LR1*^ *ubi-GFP FRT2A*

*Apc1* or *Apc2* mutant clones: *Ubx-FLP/+; FRT82B Apc1*^*Q8*^*/FRT82B arm-lacZ* or *Ubx-FLP/+; FRT82B Apc2*^*19*.*3*^*/FRT82B arm-lacZ*

### Cell culture and transfection

S2R+ cells (Drosophila Genomics Resource Center) were maintained at 25°C in Schneider’s complete medium: Schneider’s Drosophila medium with L-glutamine (Gibco) supplemented with 10% FBS (Gibco) and 0.1mg/mL penicillin/streptomycin (Invitrogen). Cells were transiently transfected using calcium-phosphate DNA precipitation [73].

### Wingless conditioned medium

To collect Wingless conditioned medium (Wg CM), S2TubWg cells (Drosophila Genomics Resource Center) were grown to confluence, then split 1:3 and incubated at 25°C for 72 hours. Cells were then re-suspended in the medium and centrifuged at 1000 x rpm for 5 minutes at room temperature; the supernatant was centrifuged again at 5000 x rpm for 5 minutes at room temperature. The resulting supernatant contained the Wingless conditioned medium, which was stored at 4°C.

For treatment with Wg CM, cells were washed one time with serum-free, antibiotic-free, Schneider’s medium. Wg CM or complete medium (CTR) was added and cells were incubated at 25°C for 1 hour.

### Transgenes and plasmids

*pUASTattB-Axin-V5* and *pUASTattB-AxinΔTBD-V5* were generated as described [18]. To generate *pUASTattB-Myr-Axin-V5* transgene, the myristoylation sequence was added to *pUASTattB-Axin-V5* [18] by two rounds of PCR-mediated mutagenesis using the oligonucleotides: forward: 5’-ATG GGC AAC AAA TGC TGC AGC AAG CGA CAG AGT TTC atg agt ggc cat cca tcg gga at-3’ (residues in upper-case denote the myristoylation sequence), and reverse: 5’-gtc aac ttc ctc gag cag-3’. The resulting PCR product was used as a template and amplified with the oligonucleotides: forward: 5’-gag ggt acc tac tag tcc agt gtg gtg gaa ttg atc ATG GGC AAC AAA TGC TGC AGC AA -3’, and reverse: 5’-gtc aac ttc ctc gag cag-3’. The resulting fragment was digested with *KpnI* and *XhoI* and inserted into *pUASTattB-Axin-V5* at the *KpnI* and *XhoI* sites.

The control *pUASTattB-Myr*^*G-A*^*-Axin-V5* was generated by PCR-mediated mutagenesis using *pUASTattB-Myr-Axin-V5* as a template and the oligonucleotides: forward: 5’- gag ggt acc tac tag tcc agt gtg gtg gaa ttg atc ATG GCC AAC AAA TGC TGC AGC AA -3’ and reverse: 5’-gtc aac ttc ctc gag cag-3’. The resulting fragment was digested with *KpnI* and *XhoI* and inserted into *pUASTattB-Axin-V5* at the *KpnI* and *XhoI* sites.

To generate *pUASTattB-Myr-AxinΔTBD-V5*, the same procedure and the same oligonucleotides as above were used. The resulting fragment was digested with *KpnI* and *XhoI* and inserted into *pUASTattB-AxinΔTBD-V5* at the *KpnI* and *XhoI* sites.

Plasmids used for transfection of Drosophila S2R+ cells were *pAc5*.*1-Axin-V5*, *pAc5*.*1-AxinΔTBD-V5*, *pAc5*.*1A-Myr-Axin-V5* and *pAc5*.*1-Myr-AxinΔTBD-V5*. To generate the plasmids: fragments encoding *Axin-V5*, *AxinΔTBD-V5*, *Myr-Axin-V5*, *Myr-AxinΔTBD-V5* from *pUASTattB-Axin-V5*, *pUASTattB-AxinΔTBD-V5*, *pUASTattB-Myr-Axin-V5* and *pUASTattB-Myr-AxinΔTBD-V5* respectively, were digested using *KpnI* and *XbaI*. The resulting fragments were inserted into the *pAc5*.*1 A* vector (Invitrogen) at the *KpnI* and *XbaI* sites.

### Immunoblots and immunostaining

For S2R+ cell lysates used in immunoblots, cells were washed with cold PBS and lysed in 4X Laemmli buffer supplemented with 0.1M DTT. For larval lysates used in immunoblots, third instar larvae were dissected to remove salivary glands, fat body, and gut tissues in cold PBS. After removal of PBS, 4X Laemmli loading buffer supplemented with 0.1M DTT was added and the lysates were vortexed briefly. For midgut lysates used in immunoblots, midguts from 5-day-old adults were dissected in cold PBS, and then treated with 1x Trypsin EDTA (Corning Life Sciences) for 2 hours at room temperature. Tissues were washed with PBS and homogenized in 4X Laemmli loading buffer supplemented with 0.1M DTT. All the lysates were incubated for 5 minutes at 100°C before SDS-PAGE analysis. Quantification of immunoblots was performed with ImageJ (Wayne Rasband, National Institutes of Health). Statistical analysis (t-test) was performed using Prism (GraphPad).

For immunostaining, third instar larval wing imaginal discs and pupal wings were dissected in PBS, fixed in 4% paraformaldehyde in PBS for 20 minutes. For immunostaining of adult guts, midguts were dissected in PBS, fixed in 4% paraformaldehyde in PBS for 45 minutes. After fixation, all samples were washed with PBS with 0.1% Triton X-100, followed by incubation in PBS with 0.5% Triton X-100 and 10% BSA for 1 hour at room temperature. Incubation with primary antibodies was performed at 4°C overnight in PBS with 0.5% Triton X-100. Incubation with secondary antibodies was for 2 hours at room temperature. Specimens were mounted in Prolong Gold (Invitrogen). Immunostaining of embryos was performed as described [18]. Fluorescent images were obtained on a Nikon A1RSi confocal microscope or Zeiss LSM 880 microscope with Airyscan (Fig 2G–2I) and processed using Adobe Photoshop software.

### Antibodies

The primary antibodies used for immunoblotting were mouse anti-V5 (1:5000, Invitrogen), guinea pig anti-Axin (1:1000, [17]), rabbit anti-Kinesin Heavy Chain (1:10000, Cytoskeleton), mouse anti-alpha-Tubulin (1:10000, DM1A, Sigma), rabbit anti-alpha-Tubulin (1:10000, Sigma), rabbit anti-Gluthathione-S-Transferase (1:10000, Invitrogen), rabbit anti-phospho-LRP6 [Thr1572] (1:1000, Millipore), mouse anti-Nervana antibody (Nrv5F7, 1:1000, DSHB), and guinea pig anti-Arrow antibody (1:1000, [74]). The primary antibodies used for immunostaining were guinea pig anti-Axin (1:1000, [17]), rabbit anti-β-gal (1:1000; MP Biomedicals), mouse anti-Arm (1:20; DSHB), mouse anti-Fas III (1:20; DSHB), mouse anti-Discs Large (1:20; DSHB), rabbit anti-GFP (1:200; Invitrogen), mouse anti-V5 (1:5000; Invitrogen), rabbit anti-Apc2 (1:1000; [75]), and guinea pig anti-Senseless (1:1000, [76]).

The secondary antibodies used for immunoblotting were: goat anti-rabbit HRP conjugate (1:10000, Biorad), goat anti-mouse HRP conjugate (1:10000, Biorad), and goat anti-guinea pig HRP conjugate (1:10000, Jackson ImmunoResearch). The secondary antibodies used for immunostaining were goat or donkey Alexa Fluor 488, 555 or Cy5 conjugates (1:400; Invitrogen).

### Subcellular fractionation

Embryos were collected 0–2.5 hours after egg lay, dechorionated in bleach for 45 seconds, and washed extensively with water and 1X PBS. Embryos and S2R+ cells were lysed in lysis buffer (20mM HEPES, 10mM KCL, pH 7.9) supplemented with 0.5mM DTT and 1X protease/phosphatase inhibitor cocktail (Pierce) with a dounce homogenizer (200 strokes). Lysates were spun at 1000 x g for 10 minutes to obtain total lysate. Supernatant was subsequently spun at 100,000 x g for 30 minutes to pellet the membrane fraction. Supernatant containing cytosolic fraction was saved and pellet containing membrane fraction was resuspended in lysis buffer supplemented with 0.5mM DTT, 1% NP-40, and 1X protease/phosphatase inhibitor cocktail (Pierce).

### GST-WWE pull-down assay

For GST pull downs, GST-WWE and GST-WWE^R164A^ beads were generated as described previously [14]. S2R+ cells were treated as indicated, then washed once with cold 1X PBS and lysed in RIPA buffer (50mM Tris [pH 8.5], 300 mM NaCl, 1% NP-40, 0.5% sodium deoxycholate, and 0.1% SDS) supplemented with 1μM ADP-HPD (Enzo Lifesciences), and 1X protease and phosphatase inhibitor cocktail (Pierce). Lysates were incubated with GST-WWE or GST-WWE^R164A^ beads overnight at 4°C. Following incubation, beads were washed four times in wash buffer (50mM TrisHCl [pH 8.0], 150mM NaCl, 1% NP-40, 10% Glycerol, 1.5mM EDTA [pH 8.0]) supplemented with 1μM ADP-HPD and 1X protease and phosphatase inhibitor cocktail (Pierce). Bound materials were eluted with 4X sample buffer and resolved by SDS-PAGE, transferred to nitrocellulose membranes and blotted with the indicated antibodies.

## Supporting Information

S1 Fig Axin membrane localization is independent of the Wingless co-receptors Arrow and Frizzled. (A-C) Confocal images of third instar larval wing discs with *arrow* null mutant clones (marked by the absence of GFP) stained with antibodies against Axin (green) and GFP (magenta). The intensity of Axin staining at the basolateral membrane is the same in wild-type and *arrow* mutant cells. To obtain *arrow* clones, mosaics of *arrow* and *PCNA* mutant cells, which are proliferation impaired, were generated using *hedgehog-Gal4*, *UAS-FLP*. (D-F) Confocal images of third instar larval wing discs with *fz Dfz2* double null mutant clones (marked by the absence of GFP) stained with antibodies against Axin (green) and GFP (magenta). The intensity of Axin staining at the basolateral membrane is the same in wild-type and *fz Dfz2* mutant cells. To obtain *fz Dfz2* clones, mosaics of *fz Dfz2* and *cyclin A* mutant cells, which are proliferation impaired, were generated with *engrailed-Gal4*, *UAS-FLP*. Scale bar: 5μm.
(TIF)

S2 Fig Expression of membrane-associated Axin within physiological range. Expressing *Axin-V5* or *Myr-Axin-V5* with the *71-Gal4* driver in larval wing discs does not disrupt expression of the Wingless target gene *senseless* (A-C), or the morphology of adult wings (D-F). Yellow arrows in (A-C) indicate the dorsoventral boundary of the larval wing disc. Boxed areas in (D-F) are shown in (D’-F’). 15–20 flies of each genotype were examined. Scale bar: 20μm.
(TIF)

S3 Fig Membrane association promotes Tnks-mediated Axin degradation in adult midguts (A) Lysates from the midguts of adult flies expressing indicated transgenes by *Myo1A-Gal4* driver were analyzed by immunoblotting. Myr-Axin-V5 was present at a much lower level compared with Axin-V5 or Myr^G-A^-Axin-V5. Kinesin was used as a loading control. (B) Lysates of the midguts of adult flies with indicated genotypes were analyzed by immunoblotting. Eliminating Tnks restores the protein levels of Myr-Axin-V5. Transgenes were expressed using *Myo1A-Gal4* driver. Kinesin was used as a loading control. (C-E) Immunostaining of the adult midguts with indicated genotype. Myr-Axin-V5 localizes predominately at the cell membrane in *Tnks* mutants where its levels are comparable to that of Axin-V5. Scale bar: 20μm.
(TIF)

S4 Fig Axin is uniformly distributed in the embryonic ectoderm prior to the onset of Wingless expression. (A-C) Confocal images of embryos expressing *Axin-V5* driven by the *mat-Gal4* driver. Embryonic stage and developmental time in hours after egg lay (AEL) are indicated at the top right of panels A. Anterior left, dorsal up. Embryos were stained with V5 and Neurotactin antibodies. Prior to the onset of Wingless expression (stage 5–6), Axin-V5 is uniformly distributed throughout the embryo. (D-F) Higher magnification images reveal that Axin-V5 partially co-localizes with the transmembrane protein Neurotactin in all ectodermal cells. Axin is also diffuse in the cytoplasm. Scale bar: 10μm.
(TIF)

S5 Fig Axin levels are increased in the cytoplasm and at the plasma membrane following Wingless exposure. Stage 9 embryos expressing the *Axin-V5* transgene were stained with V5 (A-A”) and Wg antibodies (B-B”). Axin-V5 increases both at the plasma membrane and cytoplasm (white arrows) in segmental stripes that overlap with Wg stripes (C-C”). Similar patterns were observed at different focal planes, suggesting an overall increase of Axin levels in response to Wg stimulation. Cells not exposed to Wg display weaker Axin-V5 staining both at the cell membrane and cytoplasm (yellow arrowheads). Z series images are from apical to basal levels (A-A”) at 0.5μm steps. Scale bar: 20μm.
(TIF)

## References

[1] Clevers H, Nusse R. Wnt/beta-catenin signaling and disease. Cell. 2012 6 8;149(6):1192–205. Epub 2012/06/12. eng. 10.1016/j.cell.2012.05.012 22682243

[2] MacDonald BT, Tamai K, He X. Wnt/beta-catenin signaling: components, mechanisms, and diseases. Developmental cell. 2009 7;17(1):9–26. Pubmed Central PMCID: 2861485. Epub 2009/07/22. eng. 10.1016/j.devcel.2009.06.016 19619488 PMC2861485

[3] Mao J, Wang J, Liu B, Pan W, Farr GH 3rd, Flynn C, et al Low-density lipoprotein receptor-related protein-5 binds to Axin and regulates the canonical Wnt signaling pathway. Molecular cell. 2001 4;7(4):801–9. 11336703 10.1016/s1097-2765(01)00224-6

[4] Zeng X, Huang H, Tamai K, Zhang X, Harada Y, Yokota C, et al Initiation of Wnt signaling: control of Wnt coreceptor Lrp6 phosphorylation/activation via frizzled, dishevelled and axin functions. Development. 2008 1;135(2):367–75. 10.1242/dev.013540 18077588 PMC5328672

[5] Kim SE, Huang H, Zhao M, Zhang X, Zhang A, Semonov MV, et al Wnt stabilization of beta-catenin reveals principles for morphogen receptor-scaffold assemblies. Science. 2013 5 17;340(6134):867–70. Pubmed Central PMCID: 3788643. 10.1126/science.1232389 23579495 PMC3788643

[6] Davidson G, Wu W, Shen J, Bilic J, Fenger U, Stannek P, et al Casein kinase 1 gamma couples Wnt receptor activation to cytoplasmic signal transduction. Nature. 2005 12 8;438(7069):867–72. 10.1038/nature04170 16341016

[7] Bilic J, Huang YL, Davidson G, Zimmermann T, Cruciat CM, Bienz M, et al Wnt induces LRP6 signalosomes and promotes dishevelled-dependent LRP6 phosphorylation. Science. 2007 6 15;316(5831):1619–22. 10.1126/science.1137065 17569865

[8] Cselenyi CS, Jernigan KK, Tahinci E, Thorne CA, Lee LA, Lee E. LRP6 transduces a canonical Wnt signal independently of Axin degradation by inhibiting GSK3's phosphorylation of beta-catenin. Proceedings of the National Academy of Sciences of the United States of America. 2008 6 10;105(23):8032–7. Pubmed Central PMCID: 2430354. Epub 2008/05/30. eng. 10.1073/pnas.0803025105 18509060 PMC2430354

[9] Zeng X, Tamai K, Doble B, Li S, Huang H, Habas R, et al A dual-kinase mechanism for Wnt co-receptor phosphorylation and activation. Nature. 2005 12 8;438(7069):873–7. Pubmed Central PMCID: 2100418. 10.1038/nature04185 16341017 PMC2100418

[10] Salic A, Lee E, Mayer L, Kirschner MW. Control of beta-catenin stability: reconstitution of the cytoplasmic steps of the wnt pathway in Xenopus egg extracts. Molecular cell. 2000 3;5(3):523–32. Epub 2000/07/06. eng. 10882137 10.1016/s1097-2765(00)80446-3

[11] Lee E, Salic A, Kruger R, Heinrich R, Kirschner MW. The roles of APC and Axin derived from experimental and theoretical analysis of the Wnt pathway. PLoS biology. 2003 10;1(1):E10 Pubmed Central PMCID: 212691. Epub 2003/10/14. eng. 10.1371/journal.pbio.0000010 14551908 PMC212691

[12] Huang SM, Mishina YM, Liu S, Cheung A, Stegmeier F, Michaud GA, et al Tankyrase inhibition stabilizes axin and antagonizes Wnt signalling. Nature. 2009 10 1;461(7264):614–20. 10.1038/nature08356 19759537

[13] Callow MG, Tran H, Phu L, Lau T, Lee J, Sandoval WN, et al Ubiquitin ligase RNF146 regulates tankyrase and Axin to promote Wnt signaling. PloS one. 2011;6(7):e22595 Pubmed Central PMCID: 3143158. Epub 2011/07/30. eng. 10.1371/journal.pone.0022595 21799911 PMC3143158

[14] Zhang Y, Liu S, Mickanin C, Feng Y, Charlat O, Michaud GA, et al RNF146 is a poly(ADP-ribose)-directed E3 ligase that regulates axin degradation and Wnt signalling. Nature cell biology. 2011 5;13(5):623–9. Epub 2011/04/12. eng. 10.1038/ncb2222 21478859

[15] Feng Y, Li X, Ray L, Song H, Qu J, Lin S, et al The Drosophila tankyrase regulates Wg signaling depending on the concentration of Daxin. Cellular signalling. 2014 8;26(8):1717–24. Epub 2014/04/29. eng. 10.1016/j.cellsig.2014.04.014 24768997 PMC4346149

[16] Wang Z, Tacchelly-Benites O, Yang E, Thorne CA, Nojima H, Lee E, et al Wnt/Wingless Pathway Activation Is Promoted by a Critical Threshold of Axin Maintained by the Tumor Suppressor APC and the ADP-Ribose Polymerase Tankyrase. Genetics. 2016 5;203(1):269–81. Pubmed Central PMCID: 4858779. 10.1534/genetics.115.183244 26975665 PMC4858779

[17] Wang Z, Tian A, Benchabane H, Tacchelly-Benites O, Yang E, Nojima H, et al The ADP-ribose polymerase Tankyrase regulates adult intestinal stem cell proliferation during homeostasis in Drosophila. Development. 2016 5 15;143(10):1710–20. 10.1242/dev.127647 27190037 PMC4874480

[18] Yang E, Tacchelly-Benites O, Wang Z, Randall MP, Tian A, Benchabane H, et al Wnt pathway activation by ADP-ribosylation. Nat Commun. 2016;7:11430 10.1038/ncomms11430 27138857 PMC4857404

[19] Chiang YJ, Hsiao SJ, Yver D, Cushman SW, Tessarollo L, Smith S, et al Tankyrase 1 and tankyrase 2 are essential but redundant for mouse embryonic development. PloS one. 2008;3(7):e2639 Pubmed Central PMCID: 2441437. Epub 2008/07/10. eng. 10.1371/journal.pone.0002639 18612384 PMC2441437

[20] Tian A, Benchabane H, Wang Z, Ahmed Y. Regulation of Stem Cell Proliferation and Cell Fate Specification by Wingless/Wnt Signaling Gradients Enriched at Adult Intestinal Compartment Boundaries. PLoS genetics. 2016 2;12(2):e1005822 Pubmed Central PMCID: 4742051. 10.1371/journal.pgen.1005822 26845150 PMC4742051

[21] Cliffe A, Hamada F, Bienz M. A role of Dishevelled in relocating Axin to the plasma membrane during wingless signaling. Current biology: CB. 2003 5 27;13(11):960–6. Epub 2003/06/05. eng. 12781135 10.1016/s0960-9822(03)00370-1

[22] Mendoza-Topaz C, Mieszczanek J, Bienz M. The Adenomatous polyposis coli tumour suppressor is essential for Axin complex assembly and function and opposes Axin's interaction with Dishevelled. Open biology. 2011 11;1(3):110013 Pubmed Central PMCID: 3352083. 10.1098/rsob.110013 22645652 PMC3352083

[23] Tolwinski NS, Wehrli M, Rives A, Erdeniz N, DiNardo S, Wieschaus E. Wg/Wnt signal can be transmitted through arrow/LRP5,6 and Axin independently of Zw3/Gsk3beta activity. Developmental cell. 2003 3;4(3):407–18. Epub 2003/03/15. eng. 12636921 10.1016/s1534-5807(03)00063-7

[24] Willert K, Shibamoto S, Nusse R. Wnt-induced dephosphorylation of axin releases beta-catenin from the axin complex. Genes & development. 1999 7 15;13(14):1768–73. Pubmed Central PMCID: 316878. Epub 1999/07/27. eng. 10421629 10.1101/gad.13.14.1768 PMC316878

[25] Yamamoto H, Kishida S, Kishida M, Ikeda S, Takada S, Kikuchi A. Phosphorylation of axin, a Wnt signal negative regulator, by glycogen synthase kinase-3beta regulates its stability. The Journal of biological chemistry. 1999 4 16;274(16):10681–4. Epub 1999/04/10. eng. 10196136 10.1074/jbc.274.16.10681

[26] Kofron M, Birsoy B, Houston D, Tao Q, Wylie C, Heasman J. Wnt11/beta-catenin signaling in both oocytes and early embryos acts through LRP6-mediated regulation of axin. Development. 2007 2;134(3):503–13. Epub 2007/01/05. eng. 10.1242/dev.02739 17202189

[27] Muller HA, Wieschaus E. armadillo, bazooka, and stardust are critical for early stages in formation of the zonula adherens and maintenance of the polarized blastoderm epithelium in Drosophila. The Journal of cell biology. 1996 7;134(1):149–63. Pubmed Central PMCID: 2120925. Epub 1996/07/01. eng. 8698811 10.1083/jcb.134.1.149 PMC2120925

[28] Hamada F, Tomoyasu Y, Takatsu Y, Nakamura M, Nagai S, Suzuki A, et al Negative regulation of Wingless signaling by D-axin, a Drosophila homolog of axin. Science. 1999 3 12;283(5408):1739–42. Epub 1999/03/12. eng. 10073940 10.1126/science.283.5408.1739

[29] Woods DF, Wu JW, Bryant PJ. Localization of proteins to the apico-lateral junctions of Drosophila epithelia. Developmental genetics. 1997;20(2):111–8. 10.1002/(SICI)1520-6408(1997)20:2<111::AID-DVG4>3.0.CO;2-A 9144922

[30] Werner T, Koshikawa S, Williams TM, Carroll SB. Generation of a novel wing colour pattern by the Wingless morphogen. Nature. 2010 4 22;464(7292):1143–8. 10.1038/nature08896 20376004

[31] Parnas D, Haghighi AP, Fetter RD, Kim SW, Goodman CS. Regulation of postsynaptic structure and protein localization by the Rho-type guanine nucleotide exchange factor dPix. Neuron. 2001 11 8;32(3):415–24. 11709153 10.1016/s0896-6273(01)00485-8

[32] Baker NE. Molecular cloning of sequences from wingless, a segment polarity gene in Drosophila: the spatial distribution of a transcript in embryos. The EMBO journal. 1987 6;6(6):1765–73. Pubmed Central PMCID: 553553. Epub 1987/06/01. eng. 16453776 10.1002/j.1460-2075.1987.tb02429.x PMC553553

[33] Wehrli M, Dougan ST, Caldwell K, O'Keefe L, Schwartz S, Vaizel-Ohayon D, et al arrow encodes an LDL-receptor-related protein essential for Wingless signalling. Nature. 2000 9 28;407(6803):527–30. Epub 2000/10/12. eng. 10.1038/35035110 11029006

[34] Muller HA, Samanta R, Wieschaus E. Wingless signaling in the Drosophila embryo: zygotic requirements and the role of the frizzled genes. Development. 1999 2;126(3):577–86. Epub 1999/01/07. eng. 9876186 10.1242/dev.126.3.577

[35] Chen CM, Struhl G. Wingless transduction by the Frizzled and Frizzled2 proteins of Drosophila. Development. 1999 12;126(23):5441–52. 10556068 10.1242/dev.126.23.5441

[36] Jones KH, Liu J, Adler PN. Molecular analysis of EMS-induced frizzled mutations in Drosophila melanogaster. Genetics. 1996 1;142(1):205–15. Pubmed Central PMCID: 1206949. 8770598 10.1093/genetics/142.1.205 PMC1206949

[37] McCartney BM, Dierick HA, Kirkpatrick C, Moline MM, Baas A, Peifer M, et al Drosophila APC2 is a cytoskeletally-associated protein that regulates wingless signaling in the embryonic epidermis. The Journal of cell biology. 1999 9 20;146(6):1303–18. Pubmed Central PMCID: 2156123. 10491393 10.1083/jcb.146.6.1303 PMC2156123

[38] Yu X, Waltzer L, Bienz M. A new Drosophila APC homologue associated with adhesive zones of epithelial cells. Nature cell biology. 1999 7;1(3):144–51. 10.1038/11064 10559900

[39] Simon MA, Drees B, Kornberg T, Bishop JM. The nucleotide sequence and the tissue-specific expression of Drosophila c-src. Cell. 1985 10;42(3):831–40. 2996778 10.1016/0092-8674(85)90279-x

[40] Zecca M, Basler K, Struhl G. Direct and long-range action of a wingless morphogen gradient. Cell. 1996 11 29;87(5):833–44. 8945511 10.1016/s0092-8674(00)81991-1

[41] Struhl G, Adachi A. Nuclear access and action of notch in vivo. Cell. 1998 5 15;93(4):649–60. 9604939 10.1016/s0092-8674(00)81193-9

[42] Tolwinski NS. Membrane bound axin is sufficient for Wingless signaling in Drosophila embryos. Genetics. 2009 3;181(3):1169–73. Pubmed Central PMCID: 2651051. 10.1534/genetics.108.098236 19124571 PMC2651051

[43] Kamps MP, Buss JE, Sefton BM. Mutation of NH2-terminal glycine of p60src prevents both myristoylation and morphological transformation. Proceedings of the National Academy of Sciences of the United States of America. 1985 7;82(14):4625–8. Pubmed Central PMCID: 390438. 2991884 10.1073/pnas.82.14.4625 PMC390438

[44] DaRosa PA, Wang Z, Jiang X, Pruneda JN, Cong F, Klevit RE, et al Allosteric activation of the RNF146 ubiquitin ligase by a poly(ADP-ribosyl)ation signal. Nature. 2015 1 8;517(7533):223–6. Pubmed Central PMCID: 4289021. 10.1038/nature13826 25327252 PMC4289021

[45] Wang Z, Michaud GA, Cheng Z, Zhang Y, Hinds TR, Fan E, et al Recognition of the iso-ADP-ribose moiety in poly(ADP-ribose) by WWE domains suggests a general mechanism for poly(ADP-ribosyl)ation-dependent ubiquitination. Genes & development. 2012 2 1;26(3):235–40. Pubmed Central PMCID: 3278890. 22267412 10.1101/gad.182618.111 PMC3278890

[46] Hortsch M, Patel NH, Bieber AJ, Traquina ZR, Goodman CS. Drosophila neurotactin, a surface glycoprotein with homology to serine esterases, is dynamically expressed during embryogenesis. Development. 1990 12;110(4):1327–40. 2100266 10.1242/dev.110.4.1327

[47] Luo W, Peterson A, Garcia BA, Coombs G, Kofahl B, Heinrich R, et al Protein phosphatase 1 regulates assembly and function of the beta-catenin degradation complex. The EMBO journal. 2007 3 21;26(6):1511–21. Pubmed Central PMCID: 1829374. 10.1038/sj.emboj.7601607 17318175 PMC1829374

[48] Taelman VF, Dobrowolski R, Plouhinec JL, Fuentealba LC, Vorwald PP, Gumper I, et al Wnt signaling requires sequestration of glycogen synthase kinase 3 inside multivesicular endosomes. Cell. 2010 12 23;143(7):1136–48. Pubmed Central PMCID: 3022472. 10.1016/j.cell.2010.11.034 21183076 PMC3022472

[49] Gagliardi M, Hernandez A, McGough IJ, Vincent JP. Inhibitors of endocytosis prevent Wnt/Wingless signalling by reducing the level of basal beta-catenin/Armadillo. Journal of cell science. 2014 11 15;127(Pt 22):4918–26. Pubmed Central PMCID: 4231306. 10.1242/jcs.155424 25236598 PMC4231306

[50] Thorvaldsen TE, Pedersen NM, Wenzel EM, Schultz SW, Brech A, Liestol K, et al Structure, Dynamics, and Functionality of Tankyrase Inhibitor-Induced Degradasomes. Molecular cancer research: MCR. 2015 11;13(11):1487–501. 10.1158/1541-7786.MCR-15-0125 26124443

[51] Martino-Echarri E, Brocardo MG, Mills KM, Henderson BR. Tankyrase Inhibitors Stimulate the Ability of Tankyrases to Bind Axin and Drive Assembly of beta-Catenin Degradation-Competent Axin Puncta. PloS one. 2016;11(3):e0150484 Pubmed Central PMCID: 4773256. 10.1371/journal.pone.0150484 26930278 PMC4773256

[52] Fagotto F, Jho E, Zeng L, Kurth T, Joos T, Kaufmann C, et al Domains of axin involved in protein-protein interactions, Wnt pathway inhibition, and intracellular localization. The Journal of cell biology. 1999 5 17;145(4):741–56. Pubmed Central PMCID: 2133179. 10330403 10.1083/jcb.145.4.741 PMC2133179

[53] Faux MC, Coates JL, Catimel B, Cody S, Clayton AH, Layton MJ, et al Recruitment of adenomatous polyposis coli and beta-catenin to axin-puncta. Oncogene. 2008 10 2;27(44):5808–20. 10.1038/onc.2008.205 18591934

[54] Fiedler M, Mendoza-Topaz C, Rutherford TJ, Mieszczanek J, Bienz M. Dishevelled interacts with the DIX domain polymerization interface of Axin to interfere with its function in down-regulating beta-catenin. Proceedings of the National Academy of Sciences of the United States of America. 2011 2 1;108(5):1937–42. Pubmed Central PMCID: 3033301. 10.1073/pnas.1017063108 21245303 PMC3033301

[55] de la Roche M, Ibrahim AE, Mieszczanek J, Bienz M. LEF1 and B9L shield beta-catenin from inactivation by Axin, desensitizing colorectal cancer cells to tankyrase inhibitors. Cancer research. 2014 3 1;74(5):1495–505. Pubmed Central PMCID: 3947273. 10.1158/0008-5472.CAN-13-2682 24419084 PMC3947273

[56] Pronobis MI, Rusan NM, Peifer M. A novel GSK3-regulated APC:Axin interaction regulates Wnt signaling by driving a catalytic cycle of efficient betacatenin destruction. eLife. 2015;4:e08022 Pubmed Central PMCID: 4568445. 10.7554/eLife.08022 26393419 PMC4568445

[57] Yeh TY, Meyer TN, Schwesinger C, Tsun ZY, Lee RM, Chi NW. Tankyrase recruitment to the lateral membrane in polarized epithelial cells: regulation by cell-cell contact and protein poly(ADP-ribosyl)ation. The Biochemical journal. 2006 11 1;399(3):415–25. Pubmed Central PMCID: 1615909. 10.1042/BJ20060713 16884355 PMC1615909

[58] Strigini M, Cohen SM. Wingless gradient formation in the Drosophila wing. Current biology: CB. 2000 3 23;10(6):293–300. 10744972 10.1016/s0960-9822(00)00378-x

[59] Yamazaki Y, Palmer L, Alexandre C, Kakugawa S, Beckett K, Gaugue I, et al Godzilla-dependent transcytosis promotes Wingless signalling in Drosophila wing imaginal discs. Nature cell biology. 2016 3 14. 10.1038/ncb3325 PMC4817240 26974662

[60] Parks AL, Cook KR, Belvin M, Dompe NA, Fawcett R, Huppert K, et al Systematic generation of high-resolution deletion coverage of the Drosophila melanogaster genome. Nature genetics. 2004 3;36(3):288–92. Epub 2004/02/26. eng. 10.1038/ng1312 14981519

[61] Ahmed Y, Hayashi S, Levine A, Wieschaus E. Regulation of armadillo by a Drosophila APC inhibits neuronal apoptosis during retinal development. Cell. 1998 6 26;93(7):1171–82. 9657150 10.1016/s0092-8674(00)81461-0

[62] Takacs CM, Baird JR, Hughes EG, Kent SS, Benchabane H, Paik R, et al Dual positive and negative regulation of wingless signaling by adenomatous polyposis coli. Science. 2008 1 18;319(5861):333–6. 10.1126/science.1151232 18202290

[63] Jafar-Nejad H, Andrews HK, Acar M, Bayat V, Wirtz-Peitz F, Mehta SQ, et al Sec15, a component of the exocyst, promotes notch signaling during the asymmetric division of Drosophila sensory organ precursors. Developmental cell. 2005 9;9(3):351–63. 10.1016/j.devcel.2005.06.010 16137928

[64] Brand AH, Perrimon N. Targeted gene expression as a means of altering cell fates and generating dominant phenotypes. Development. 1993 6;118(2):401–15. 8223268 10.1242/dev.118.2.401

[65] Vincent JP, Girdham CH, O'Farrell PH. A cell-autonomous, ubiquitous marker for the analysis of Drosophila genetic mosaics. Developmental biology. 1994 7;164(1):328–31. 10.1006/dbio.1994.1203 8026635

[66] Vincent JP, Kolahgar G, Gagliardi M, Piddini E. Steep differences in wingless signaling trigger Myc-independent competitive cell interactions. Developmental cell. 2011 8 16;21(2):366–74. Pubmed Central PMCID: 3209557. Epub 2011/08/16. eng. 10.1016/j.devcel.2011.06.021 21839923 PMC3209557

[67] Bazigou E, Apitz H, Johansson J, Loren CE, Hirst EM, Chen PL, et al Anterograde Jelly belly and Alk receptor tyrosine kinase signaling mediates retinal axon targeting in Drosophila. Cell. 2007 3 9;128(5):961–75. Epub 2007/03/14. eng. 10.1016/j.cell.2007.02.024 17350579

[68] Karpowicz P, Zhang Y, Hogenesch JB, Emery P, Perrimon N. The circadian clock gates the intestinal stem cell regenerative state. Cell reports. 2013 4 25;3(4):996–1004. Pubmed Central PMCID: 3982394. Epub 2013/04/16. eng. 10.1016/j.celrep.2013.03.016 23583176 PMC3982394

[69] Golic KG, Lindquist S. The FLP recombinase of yeast catalyzes site-specific recombination in the Drosophila genome. Cell. 1989 11 3;59(3):499–509. Epub 1989/11/03. eng. 2509077 10.1016/0092-8674(89)90033-0

[70] Hacker U, Perrimon N. DRhoGEF2 encodes a member of the Dbl family of oncogenes and controls cell shape changes during gastrulation in Drosophila. Genes & development. 1998 1 15;12(2):274–84. Pubmed Central PMCID: 316438. Epub 1998/03/07. eng. 9436986 10.1101/gad.12.2.274 PMC316438

[71] Benton R, St Johnston D. Drosophila PAR-1 and 14-3-3 inhibit Bazooka/PAR-3 to establish complementary cortical domains in polarized cells. Cell. 2003 12 12;115(6):691–704. Epub 2003/12/17. eng. 14675534 10.1016/s0092-8674(03)00938-3

[72] Xu T, Rubin GM. Analysis of genetic mosaics in developing and adult Drosophila tissues. Development. 1993 4;117(4):1223–37. 8404527 10.1242/dev.117.4.1223

[73] Graham FL, van der Eb AJ. A new technique for the assay of infectivity of human adenovirus 5 DNA. Virology. 1973 4;52(2):456–67. 4705382 10.1016/0042-6822(73)90341-3

[74] Marois E, Mahmoud A, Eaton S. The endocytic pathway and formation of the Wingless morphogen gradient. Development. 2006 1;133(2):307–17. Epub 2005/12/16. eng. 10.1242/dev.02197 16354714

[75] Ahmed Y, Nouri A, Wieschaus E. Drosophila Apc1 and Apc2 regulate Wingless transduction throughout development. Development. 2002 4;129(7):1751–62. 11923210 10.1242/dev.129.7.1751

[76] Nolo R, Abbott LA, Bellen HJ. Senseless, a Zn finger transcription factor, is necessary and sufficient for sensory organ development in Drosophila. Cell. 2000 8 4;102(3):349–62. 10975525 10.1016/s0092-8674(00)00040-4

