# Supplementary figures and images for: Dual Roles for Membrane Association of Drosophila Axin in Wnt Signaling
Source: PLoS Genet. 2016 Dec 13;12(12):e1006494. doi: 10.1371/journal.pgen.1006494 (PMC5154497; doi:10.1371/journal.pgen.1006494)

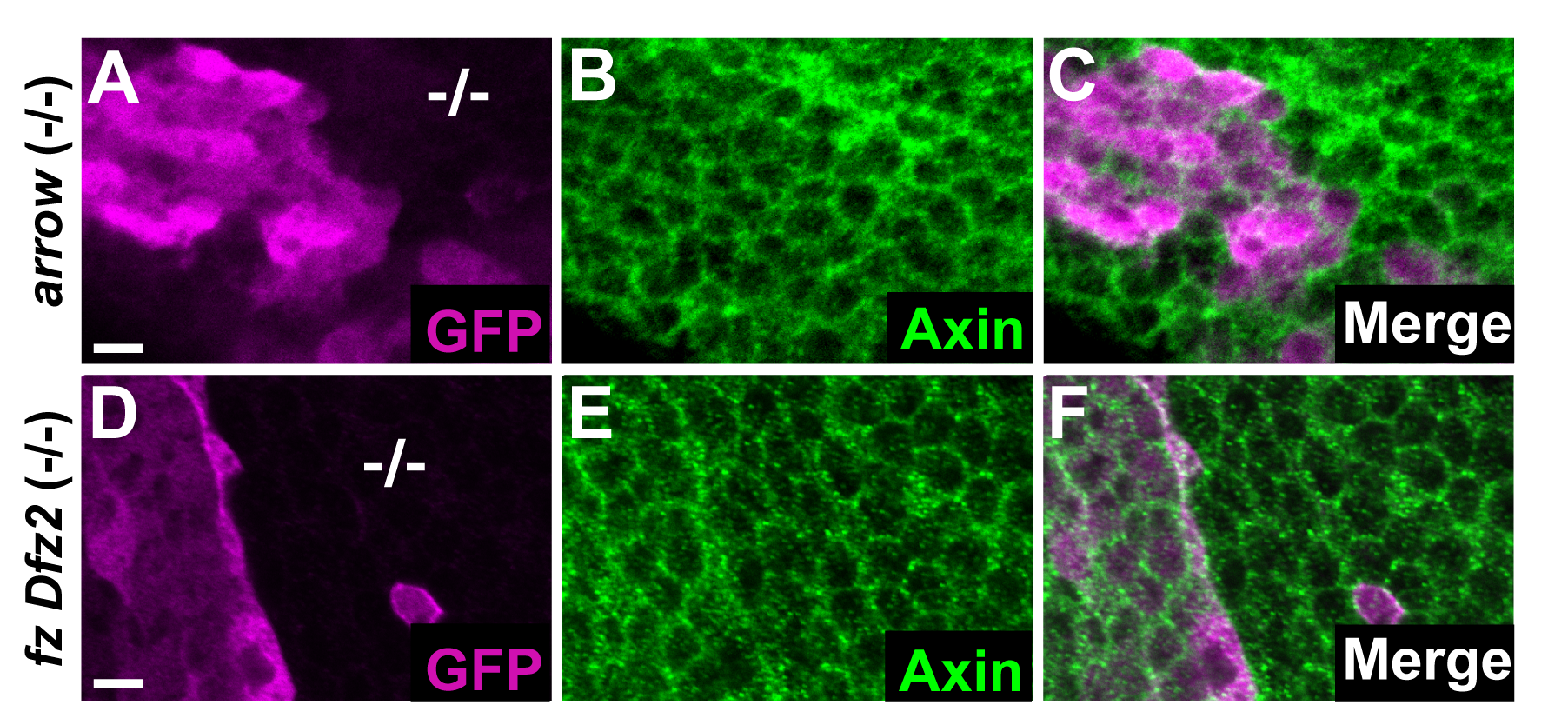

Supplement: S1 Fig — (A-C) Confocal images of third instar larval wing discs with arrow null mutant clones (marked by the absence of GFP) stained with antibodies against Axin (green) and GFP (magenta). The intensity of Axin staining at the basolateral membrane is the same in wild-type and arrow mutant cells. To obtain arrow clones, mosaics of arrow and PCNA mutant cells, which are proliferation impaired, were generated using hedgehog-Gal4, UAS-FLP. (D-F) Confocal images of third instar larval wing discs with fz Dfz2 double null mutant clones (marked by the absence of GFP) stained with antibodies against Axin (green) and GFP (magenta). The intensity of Axin staining at the basolateral membrane is the same in wild-type and fz Dfz2 mutant cells. To obtain fz Dfz2 clones, mosaics of fz Dfz2 and cyclin A mutant cells, which are proliferation impaired, were generated with engrailed-Gal4, UAS-FLP. Scale bar: 5μm. (TIF) [file pgen.1006494.s001.tif]

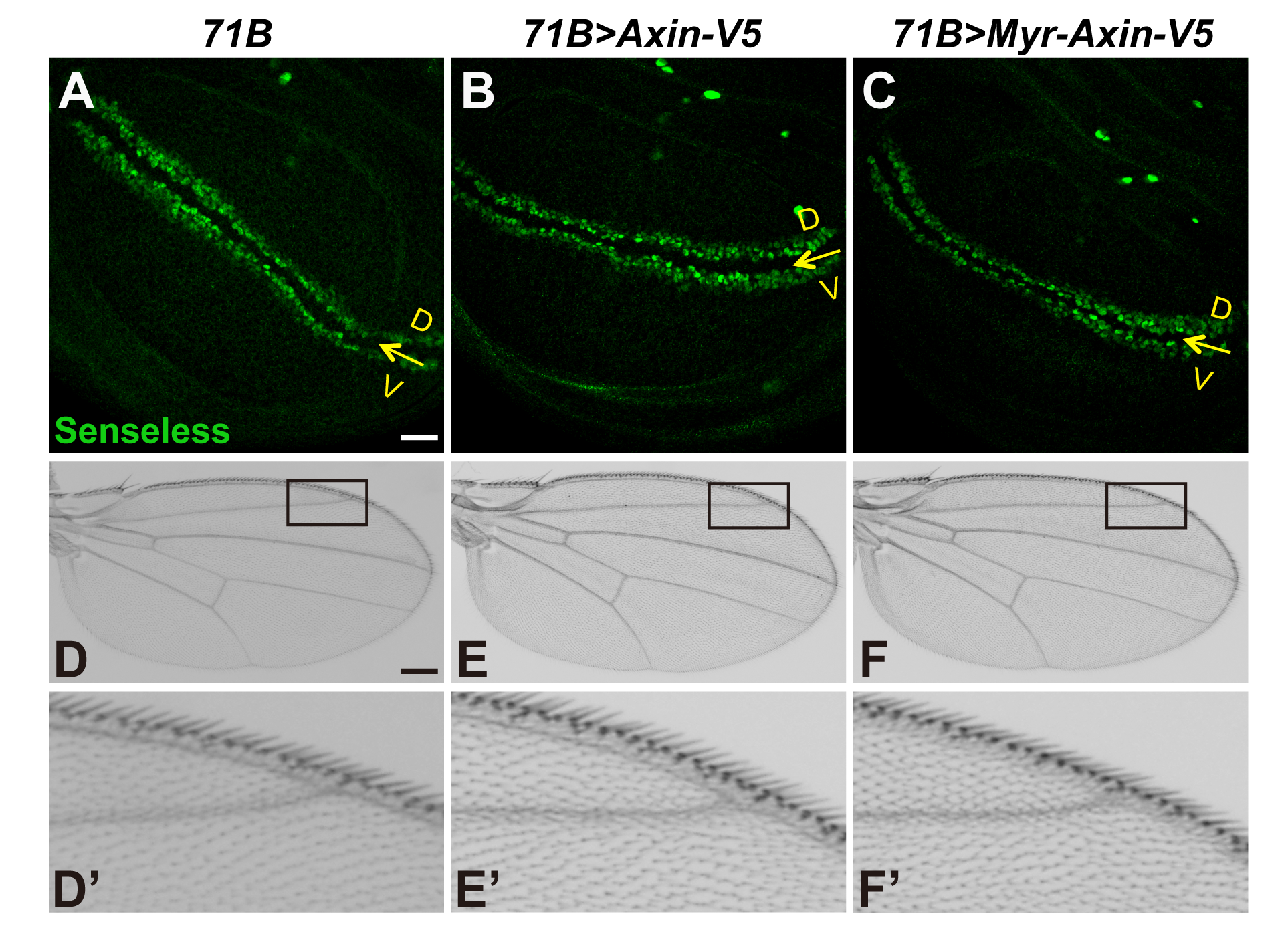

Supplement: S2 Fig — Expressing Axin-V5 or Myr-Axin-V5 with the 71-Gal4 driver in larval wing discs does not disrupt expression of the Wingless target gene senseless (A-C), or the morphology of adult wings (D-F). Yellow arrows in (A-C) indicate the dorsoventral boundary of the larval wing disc. Boxed areas in (D-F) are shown in (D’-F’). 15–20 flies of each genotype were examined. Scale bar: 20μm. (TIF) [file pgen.1006494.s002.tif]

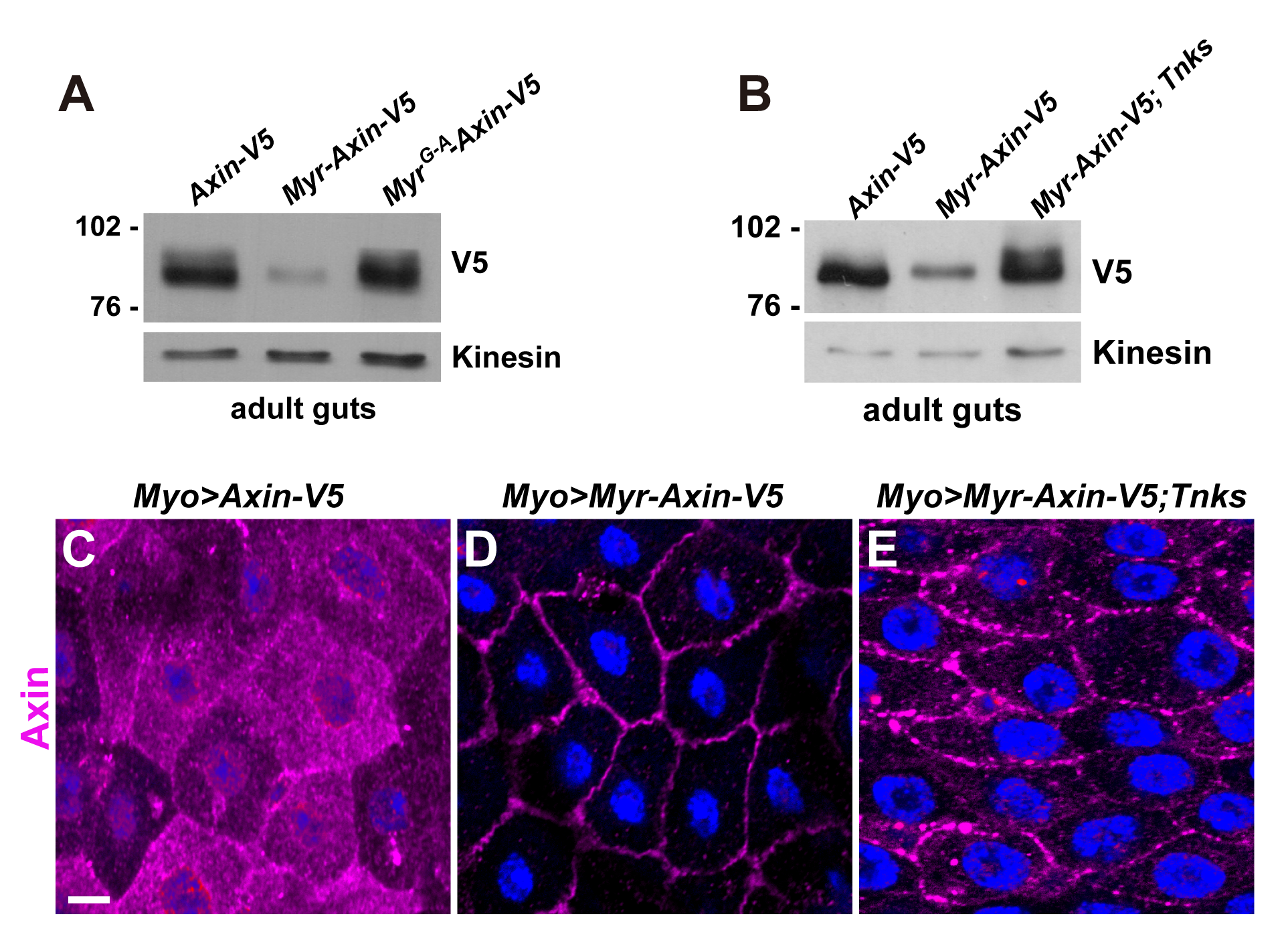

Supplement: S3 Fig — (A) Lysates from the midguts of adult flies expressing indicated transgenes by Myo1A-Gal4 driver were analyzed by immunoblotting. Myr-Axin-V5 was present at a much lower level compared with Axin-V5 or MyrG-A-Axin-V5. Kinesin was used as a loading control. (B) Lysates of the midguts of adult flies with indicated genotypes were analyzed by immunoblotting. Eliminating Tnks restores the protein levels of Myr-Axin-V5. Transgenes were expressed using Myo1A-Gal4 driver. Kinesin was used as a loading control. (C-E) Immunostaining of the adult midguts with indicated genotype. Myr-Axin-V5 localizes predominately at the cell membrane in Tnks mutants where its levels are comparable to that of Axin-V5. Scale bar: 20μm. (TIF) [file pgen.1006494.s003.tif]

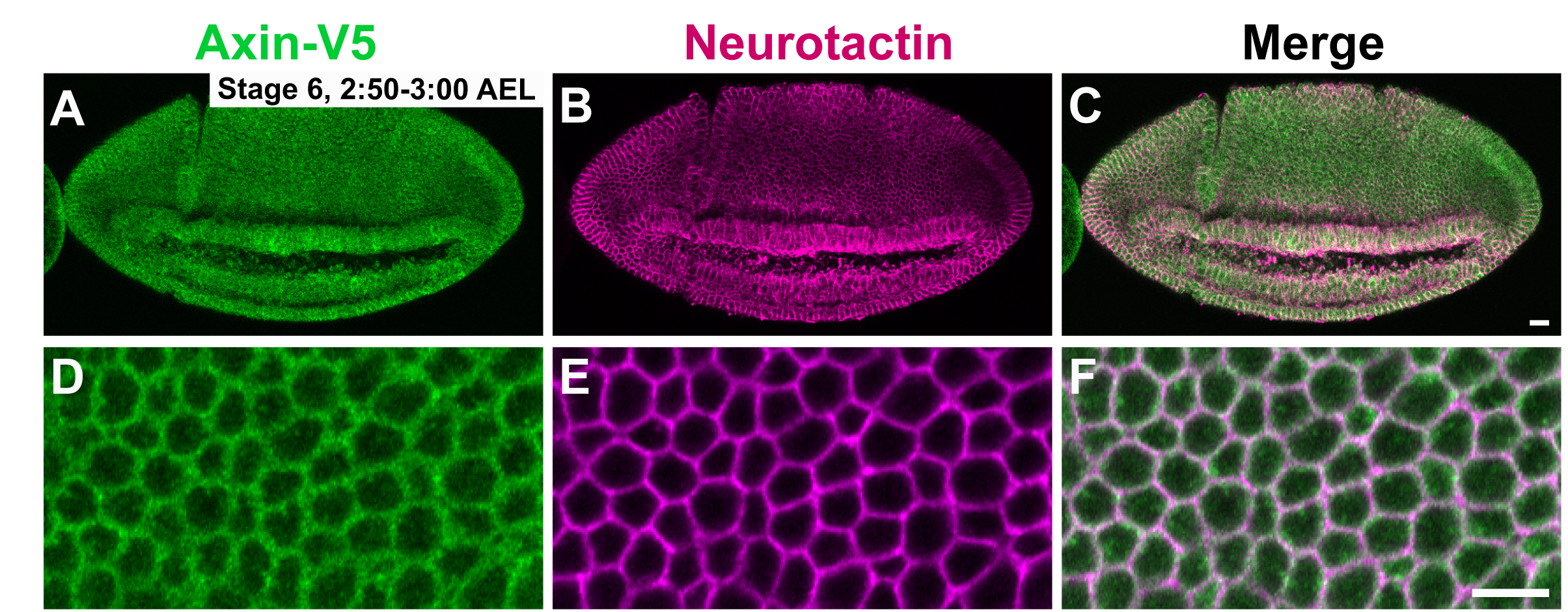

Supplement: S4 Fig — (A-C) Confocal images of embryos expressing Axin-V5 driven by the mat-Gal4 driver. Embryonic stage and developmental time in hours after egg lay (AEL) are indicated at the top right of panels A. Anterior left, dorsal up. Embryos were stained with V5 and Neurotactin antibodies. Prior to the onset of Wingless expression (stage 5–6), Axin-V5 is uniformly distributed throughout the embryo. (D-F) Higher magnification images reveal that Axin-V5 partially co-localizes with the transmembrane protein Neurotactin in all ectodermal cells. Axin is also diffuse in the cytoplasm. Scale bar: 10μm. (TIF) [file pgen.1006494.s004.tif]

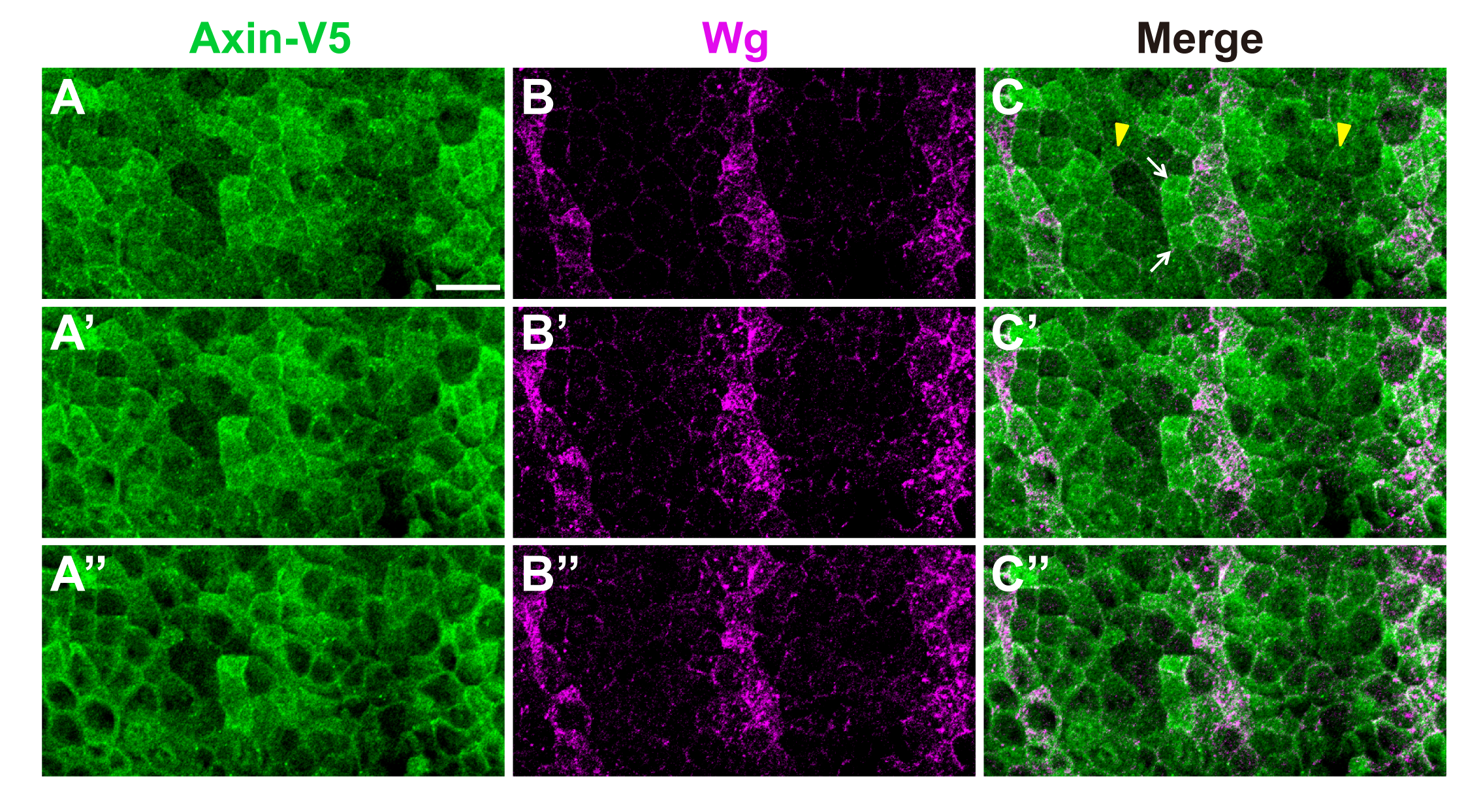

Supplement: S5 Fig — Stage 9 embryos expressing the Axin-V5 transgene were stained with V5 (A-A”) and Wg antibodies (B-B”). Axin-V5 increases both at the plasma membrane and cytoplasm (white arrows) in segmental stripes that overlap with Wg stripes (C-C”). Similar patterns were observed at different focal planes, suggesting an overall increase of Axin levels in response to Wg stimulation. Cells not exposed to Wg display weaker Axin-V5 staining both at the cell membrane and cytoplasm (yellow arrowheads). Z series images are from apical to basal levels (A-A”) at 0.5μm steps. Scale bar: 20μm. (TIF) [file pgen.1006494.s005.tif]
